# The therapeutic effect of KSP inhibitors in preclinical models of cholangiocarcinoma

**DOI:** 10.1038/s41419-022-05247-0

**Published:** 2022-09-19

**Authors:** Yuanyuan Shi, Xiaowen Cui, Tianyi Jiang, Yufei Pan, Yunkai Lin, Xiaofan Feng, Zhiwen Ding, Chun Yang, Yexiong Tan, Hongyang Wang, Liwei Dong

**Affiliations:** 1 International Cooperation Laboratory on Signal Transduction, Eastern Hepatobiliary Surgery Hospital, the Naval Medical University, Shanghai, 200438 China; 2 the First Affiliated Hospital of the Naval Medical University, Shanghai, 200433 China; 3 National Center for Liver Cancer, the Naval Medical University, Shanghai, 201805 China; 4 Department of Hepatic Surgery, Fudan University Shanghai Cancer Center, Shanghai, 200032 China; 5 Children’s Hospital of Soochow University, Suzhou, 215025 China

**Keywords:** Chemotherapy, Bile duct cancer

## Abstract

Cholangiocarcinoma (CCA) is an epithelial malignancy with a dismal prognosis owing to limited treatment options. Here, we identified several compound candidates against CCA using a high-throughput drug screen with approved or emerging oncology drugs, among which kinesin spindle protein (KSP) inhibitors showed potent cytotoxic effects on CCA cells. Treatment with KSP inhibitors SB743921 and ARRY520 caused significant tumor suppression in CCA xenograft models in vivo. Mechanistically, KSP inhibitors led to the formation of abnormal monopolar spindles, which further resulted in the mitotic arrest and cell death of CCA cells both in vivo and in vitro. KEGG pathway analysis of transcriptional data confirmed this finding. Moreover, our clinical data as well as the TCGA database showed *KIF11* expression was abundant in most CCA tumor specimens and associated with poor outcomes of CCA patients. Our results demonstrate that the therapeutic regimen of KSP inhibitors could be a promising treatment strategy in CCA.

## Introduction

Cholangiocarcinomas (CCA) are diverse biliary epithelial malignancies involving the intrahepatic, perihilar, and distal biliary tree [1, 2]. CCA is the second most common liver cancer after hepatocellular carcinoma (HCC), and the overall incidence and mortality of CCA have progressively increased worldwide over the past four decades [3–5]. CCA are aggressive tumors, and most patients have advanced-stage disease at initial diagnosis [6]. Surgery is the preferred treatment option for all CCA, but only a minority of patients (approximately 35%) with early-stage disease have the curative indication to receive surgical resection [7, 8]. For patients with advanced or unresectable CCA, the available systemic therapies present limited effectiveness: the objective response rate of the current first-line chemotherapy regimen (gemcitabine and cisplatin) is less than 30% and the median overall survival is merely 11.7 months (ABC-02 trial) [9, 10]. Albumin-bound paclitaxel has also been an option for standard-of-care regimens, but triple-agent chemotherapy combinations consisting of gemcitabine, cisplatin, and paclitaxel are currently under investigation [11]. In addition, the role of second-line chemotherapy, targeted therapy, and immunotherapy after progression on first-line chemotherapy remains unclear. Unfortunately, almost all patients inevitably experience disease progression following first-line systemic therapy and succumb to their disease. Therefore, the identification of novel and effective treatment strategies for patients with advanced CCA is urgently needed.

CCA exhibits relatively few druggable targets, though novel molecular targets such as fibroblast growth factor receptor-2 (FGFR2) and isocitrate dehydrogenase-1 (IDH1) mutations have been identified as promising within phase 2/3 trials in CCA patients harboring such alterations [12–14]. The efficacy of targeted therapies is comprised due to the highly desmoplastic nature and genetic heterogeneity of CCA, suggesting that a broader treatment strategy may be needed to combine with specific targeted therapies to completely eradicate tumors and prevent acquired resistance. High-throughput drug screens have been widely used to identify effective compounds in various cancer types [15–17]. To identify novel effective therapies for CCA, here we performed a high-throughput drug screen on CCA cells using a chemical library containing 155 approved or emerging oncology drugs. We identified several previously untested drugs with significant therapeutic effects on CCA cells, including the proteasome inhibitors, histone deacetylase (HDAC) inhibitors, and mitotic inhibitors. Our previous studies have demonstrated the proteasome inhibitors bortezomib and carfilzomib, which are prevalently used in treating multiple myeloma [18], exerted profound anti-tumor activity for PTEN-deficient CCA and have potent implications for clinical translation [19]. In this work, we focus on the mitotic inhibitors, especially the highly selective inhibitor of kinesin spindle protein (KSP, also known as Eg5).

KSP is specifically expressed by mitotic cells, responsible for the proper separation of spindle poles during cell mitosis [20–22]. Bioinformatics analysis of the TCGA dataset revealed that most of the cancer types have a relatively high level of KSP expression to support their proliferation priority. Correspondently, chemical inhibition of KSP has emerged as an appealing therapeutic strategy in the treatment of various types of tumors, such as leukemia, multiple myeloma, lymphoma, and solid tumors [23–26]. However, little is known about KSP expression and the potential effect of KSP inhibitors on CCA. Here, we identified KSP inhibitors as promising therapeutic regimens for CCA. Using the KSP inhibitor SB743921 and ARRY520, we showed that inhibiting KSP in CCA led to the formation of abnormal spindles, which resulted in mitotic arrest and apoptosis in vitro, as well as tumor regression in mouse models.

## Results

### Identification of drugs with anti-cholangiocarcinoma effect

**Fig. 1:**
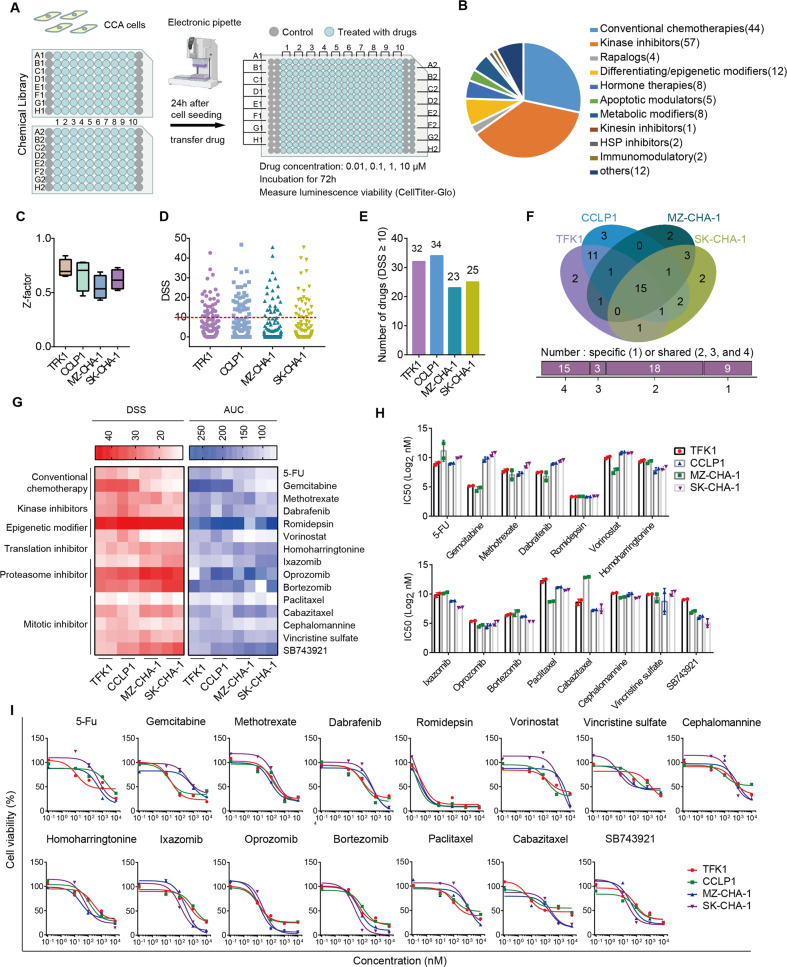
A high-throughput drug screen to identify drugs targeting CCA. **A** Design of the high-throughput drug screen. Four CCA cell lines (TFK1, CCLP1, SK-CHA-1, MZ-CHA-1) were seeded into 384-well plates and treated with drugs in the chemical library for 72 h. Experiments were performed at four concentrations per drug (10, 1, 0.1, 0.01 μM) and two replicates were set for each treatment. **B** Categories of compounds in the chemical library used for the screen. The number of each drug was shown in the fan chart. **C** The Z-factors of cell viability for each plate of CCA cell lines. The ends of the box are the upper and lower quartiles, vertical lines inside the box show the median, and whiskers mark the minimum and maximum Z-factors for each plate of CCA cell lines. **D** The DSS (drug sensitivity score) for each drug in the four CCA cell lines. **E** The number of drugs with a DSS ≥ 10 in four CCA cell lines. **F** Venn diagram of identified drugs with DSS ≥ 10 in four CCA cell lines. 15 drugs were identified with DSS ≥ 10 in all four cell lines. **G** The DSS and AUC (area under the drug-response curve) of drugs with DSS ≥ 10 in all four cell lines. **H** IC_50_ values of the 15 drugs in four cell lines. **I** Dose-response curves for the effect of 15 sensitive drugs on cell viability.

To explore the potential drugs, we used four CCA cell lines for an untargeted high-throughput drug screen, including TFK1, CCLP1, MZ-CHA-1, and SK-CHA-1. Cells were seeded in 384-well plates and incubated with series concentrations of drugs for 72 h (Fig. 1A). The screen used a library of 155 approved or emerging investigational drugs belonging to diverse drug classes, which were dispensed in 96-well plates and tested at four concentrations in 10-fold dilutions (Fig. 1B and Supplementary Data File 1). We analyzed the drug response using the CellTiter-Glo luminescent cell viability assay to determine the number of cells in each well. To assess the robustness of the assay, we calculated Z-factors for the difference in viability at each cell density using dimethyl sulfoxide (DMSO) as a negative control and the antiseptic benzethonium chloride as a positive control. We discovered that all four cell lines at four densities displayed a Z-factor > 0.5, the threshold for a robust difference (Fig. 1C).

In the screening, each drug was tested at four concentrations and produced totaling 4,960 measurement points (155 drugs × 4 concentrations × 8 parameters, Supplementary Data File 1). Concentrations producing a 50% reduction in viability (half-maximal inhibitory concentration, IC_50_), dose-response curves, and area under the curves (AUC) were calculated from the response at each concentration (Fig. S1). Moreover, these data were used to calculate a drug sensitivity score (DSS) as previously described [27] (Fig. S2), and the cut-off value was defined as 10, above which the drug was considered effective (Fig. 1D, E). To validate drugs with broad activity against CCA, we took the intersection of all selected compounds with a DSS ≥ 10 in four CCA cell lines and successfully identified 15 drugs: 3 drugs were conventional chemotherapeutics, 1 was kinase inhibitor, 2 were epigenetic modifiers, 1 was translation inhibitor, 3 were proteasome inhibitors, and 5 were mitotic inhibitors (Fig. 1F, G). Some of these drugs have been previously identified as efficient in CCA patients, such as gemcitabine and paclitaxel. Some have already shown promising activity in clinical trials, among which 5-FU has been recommended as second-line chemotherapy in advanced CCA, combined with folinic acid and oxaliplatin (FOLFOX) in phase 3 trials [28]. Dabrafenib, an inhibitor of BRAF^V600E^, also has been investigated in patients with BRAF-mutated biliary tract cancers [29]. Furthermore, our previous studies have confirmed the effectiveness of the proteasome inhibitor bortezomib in CCA [19]. Nevertheless, most of the identified compounds which have not been extensively validated in CCA, including the KSP inhibitor SB743921 (a novel mitotic inhibitor), showed potent cytotoxic effects on CCA cells with a less than 1000 nM IC_50_ value (Fig. 1H, I).

### Inhibition of cell viability in CCA cells treated with SB743921

**Fig. 2:**
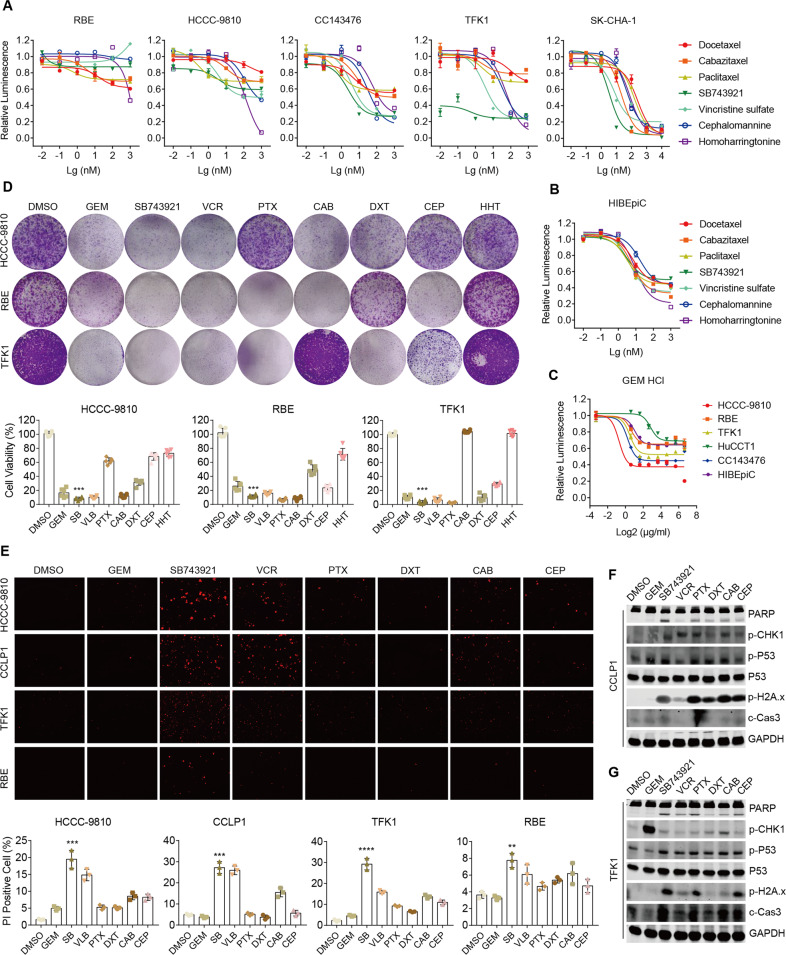
The therapeutic effect of SB743921 on CCA cell lines. **A**, **B** Cell sensitivity to drugs treatment. CCA cell line HCCC-9810, RBE, CC143476, TFK1, SK-CHA-1, and normal counterpart cell line HIBEpiC were treated with drugs at gradient concentrations (0.001, 0.01, 0.1, 1 μM) for 72 h. Values shown in the chart are the mean percent viability of control ± SD obtained from three independent experiments. **C** Cell sensitivity to gemcitabine treatment. **D** Colony formation assay of HCCC-9810, RBE, and TFK1 cells after drugs treatment (0.01 μM, 7 days). Drugs were paclitaxel (PTX), docetaxel (DXT), cabazitaxel (CAB), vincristine (VCR), and cephalomannine (CEP), homoharringtonine (HHT) or gemcitabine (GEM). Bar graphs show representative data from three independent experiments. **E** Cell viability assessment of HCCC-9810, CCLP1, TFK1, and RBE cells treated with drugs (0.25 μM, 24 h) by PI staining. Bar graphs show the mean percentage of PI-positive cells from three independent experiments. **F**, **G** Immunoblot analyses of the expression of PARP, p-CHK1, p-P53, p-H2A.x, and c-Caspase3 in CCLP1 and TFK1 cells after drugs treatment (0.25 μM, 24 h).

In drug screening assays, we discovered that mitotic inhibitors targeting microtubules were within the top effective agents, including 5 conventional drugs (paclitaxel, docetaxel, cabazitaxel, vincristine sulfate, and cephalomannine) and one novel alternative, the KSP inhibitor SB743921. We focused on mitotic inhibitors because albumin-bound paclitaxel has been recommended as a first-line option for CCA treatment, suggesting that its analogs have promising potential applications. To evaluate the cytotoxic effects of mitotic inhibitors in CCA, we carried out cell viability assays to determine the dose-response relationship in five CCA cell lines (Fig. 2A). The results showed the HCCC-9810 and RBE were resistant to all mitotic inhibitors, while CC143476, TFK1, and SK-CHA-1 were sensitive. Importantly, among all mitotic inhibitors in the sensitive cell lines, the KSP inhibitor SB743921 was the most effective agent. Cytotoxic effects of those drugs on human intrahepatic biliary epithelial cells (HIBEpiC) were also detected by cell viability assessment. Compared to CC143476, TFK1, and SK-CHA-1, HIBEpiC showed lower sensitivity to SB743921 (Fig. 2B). Moreover, the IC_50_ of SB743921 was much less than that of gemcitabine in the aforementioned sensitive cells (Fig. 2C), indicating a strong potential of clinical application of SB743921. Consistently, colony formation assays and cell viability assessment by propidium iodide (PI) staining confirmed the outstanding cytotoxic effect of SB743921 among conventional compounds in diverse CCA cell lines (Fig. 2D, E). Further immunoblots revealed that SB743921 treatment induced an obvious increase in the expression of PARP cleavage, phospho-p53, phospho-H2A.x, and cleaved caspase-3 in CCLP1 and TFK1 cells, which supported the apoptosis-promoting activity of SB743921 (Fig. 2F, G). Taken together, our data indicated that compared with gemcitabine and other conventional mitotic inhibitors, the KSP inhibitor SB743921 had a more intense therapeutic effect in CCA cells.

### Association of *KIF11* expression with prognosis and drug response in CCA

**Fig. 3:**
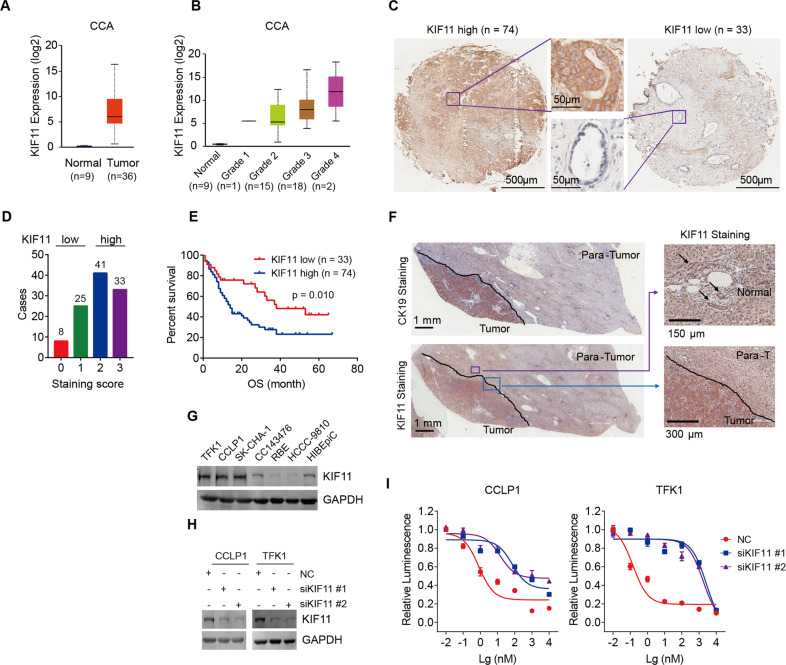
*KIF11* gene expression and dependency in CCA. **A** *KIF11* expression in CCA patient samples compared to that in normal tissue samples within the TCGA dataset. Statistical analysis was performed using the two-sided unpaired *t-*test. **B** *KIF11* expression in CCA patient samples grouped based on pathological grade. Statistical analysis was performed using the two-sided unpaired *t-*test. **C** Representative images of *KIF11* high- and low-expression in human CCA tumor tissue samples by immunohistochemical analysis. **D** The number of cases in the *KIF11* low and high groups. Human CCA samples with staining scores of 0 and 1 were allocated to the *KIF11* low-expression group (*n* = 33), whereas those with a score of 2 and 3 were allocated to the *KIF11* high-expression group (*n* = 74). **E** Overall survival of CCA patients with high and low *KIF11* gene expression using Kaplan-Meier survival curves. Statistical analyses were performed using the log-rank test. **F** Representative images of *KIF11* expression in tumor, para-tumor, and normal tissues from CCA patients by immunohistochemical analysis. Arrows indicate intrahepatic bile ducts. **G** Expression of *KIF11* in CCA cell lines and normal cell line HIBEpiC detected by immunoblotting. **H** The knockdown efficacy of *KIF11* siRNA detected by immunoblotting. **I** Cell sensitivity to SB743921 treatment in control and *KIF11*-knockdown cells. CCLP1 and TFK1 cells transfected with indicated siRNA or NC were exposed to SB743921 for 72 h. Cell viability was measured using the CellTiter-Glo luminescent cell viability assay.

Next, we assessed the correlation between differentially expressed KSP-encoding gene *KIF11* and clinical prognosis using the Human Protein Atlas (HPA) database, and the results showed that high expression of *KIF11* was associated with dismal overall survival in four cancer types, including renal cancer, liver cancer (hepatocellular carcinoma and intrahepatic cholangiocarcinoma), pancreatic cancer, and lung cancer (Fig. S3A). We further investigated the dependency of the *KIF11* gene in various cancer types using the CERES dependency score, which was based on CRISPR screening data of hundreds of cancer cell lines from publicly available datasets [30]. A lower CERES score indicates a higher dependency of the gene of interest in the given cell line. CCA cell lines presented with a higher dependency score across 27 kinds of cancer types, suggesting that CCA was vulnerable to KSP antagonism (Fig. S3B). Analysis of *KIF11* in the CCA cohort derived from the TCGA database showed higher *KIF11* expression in CCA tumors compared to that in their normal counterparts (Fig. 3A). Notably, the *KIF11* mRNA expression elevated with the increasing histological grade of CCA from the TCGA database, suggesting *KIF11* expression may correlate with CCA stage/type (Fig. 3B). We next examined *KIF11* expression in 107 tumor specimens from a human CCA cohort. According to the immunochemistry staining score, CCA tumors could be divided into a *KIF11* high-expression group or a low-expression group, with the majority of cases belonging to the *KIF11* high-expression group (Fig. 3C, D). The survival analysis showed that *KIF11* high-expression was a significant unfavorable factor for CCA prognosis, companying with a much shorter overall survival (Fig. 3E). However, neither a significant association between *KIF11* expression and histologic grade nor a significant correlation between *KIF11* expression and CCA aggressiveness was observed in our human CCA cohort (Fig. S3C).

KSP proteins are specifically expressed in mitotic cells [20–22]. Immunohistochemistry (IHC) staining showed that *KIF11* expression was higher in CCA tumors compared to para-tumor (liver) and normal (intrahepatic bile duct) tissues (Fig. 3F). Subsequently, we determined KSP expression in HIBEpiC and diverse CCA cell lines, including TFK1, CCLP1, SK-CHA-1, CC143476, RBE, and HCCC-9810 (Fig. 3G). TFK1, CCLP1, and SK-CHA-1 cells displayed a high level of *KIF11* expression, while RBE and HCCC-9810 cells exhibited lower *KIF11* expression, which might contribute to their resistance to SB743921 treatment (Fig. 2A). Knockdown of *KIF11* by two independent siRNAs (siKIF11 #1 and #2) notably decreased sensitivity of SB743921 in TFK1 and CCLP1 cells (Fig. 3H, I), which further confirmed that the growth inhibitory effect of SB743921 depended on *KIF11* expression.

### Mitotic arrest induced by SB743921 treatment

**Fig. 4:**
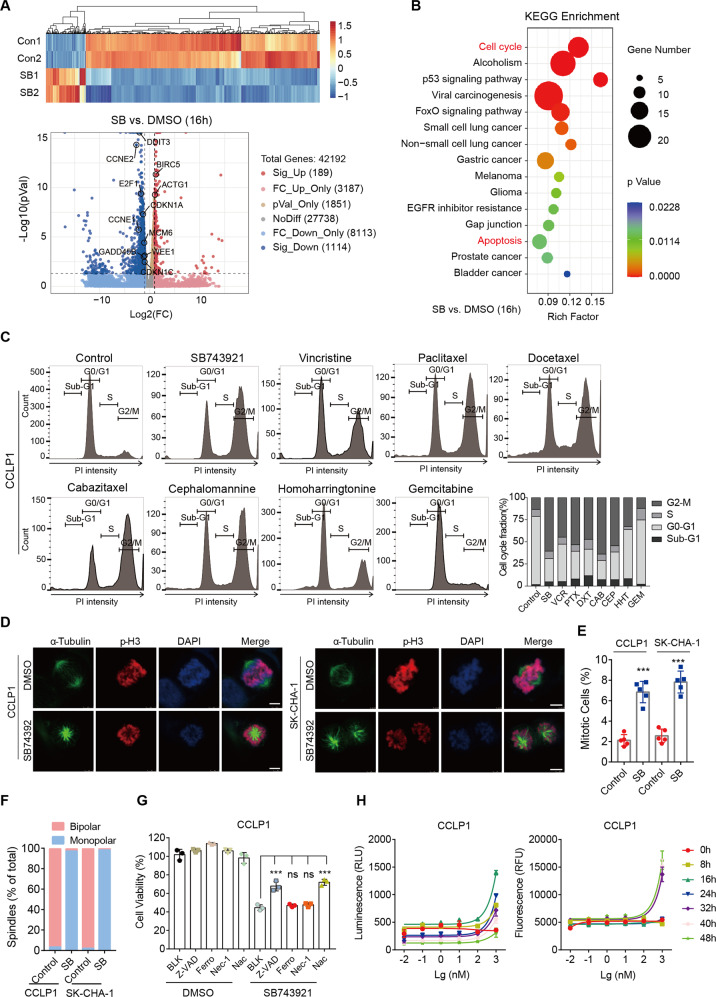
KSP inhibition causes mitotic arrest and cell apoptosis. **A** Heatmap and volcano plot of RNA-seq data from CCLP1 cells treated with SB743921 (10 nM, 16 h). Gene-expression alteration in the treated group was normalized by corresponding DMSO control. Significant genes were determined by Student’s *t* test and a threshold cutoff of *q* < 0.05, 2-fold change. Red, induced; green, repressed. **B** KEGG pathway analysis of genes affected by SB743921 treatment (10 nM, 16 h). **C** Cell cycle assessment by flow cytometry analysis. CCLP1 cells were treated with SB743921 (SB), paclitaxel (PTX), docetaxel (DXT), cabazitaxel (CAB), vincristine (VCR), cephalomannine (CEP), homoharringtonine (HHT) or gemcitabine (GEM) at a dose of 0.5 μM for 24 h. The histograms showed the fraction of cells in cell cycle phases. **D** Immunofluorescence of CCLP1 cells treated with SB743921 (0.5 μM, 24 h) and stained with α-Tubulin (green), p-H3 (red), and DAPI (blue). Scale bars, 10 μm. **E** Quantification of mitotic CCA cells after SB743921 treatment. *P* values were determined by two-tailed *t* test. **F** The fraction of bipolar and monopolar spindles in CCLP1 and SK-CHA-1 cells treated with SB743921 or DMSO. *P* values were determined by two-tailed *t* test. **G** Cell viability of CCLP1 cells treated with SB743921 alone or in combination with Z-VAD-FMK (Z-VAD, 50 μM), Ferrostatin-1 (Ferro, 20 μM), Necrostatin-1 (Nec-1, 20 μM) or N-Acetyl cysteine (Nac, 1 mM). *P* values were determined by one-way ANOVA. **H** Analysis of the type of cell death. CCLP1 cells were exposed to serial dilutions of SB743921 in the presence of the RealTime-Glo Annexin V Apoptosis and Necrosis Assay Reagent. Luminescence signal (RLU) represents apoptosis, and fluorescence signal (RFU) represents necrosis.

To further elucidate the mechanism of KSP inhibition in CCA, we performed RNA sequencing (RNA-seq) on CCLP1 cells after treatment with SB743921 (10 nM) for 8 and 16 h. Among them, 16 h RNA-seq identified 189 upregulated and 1114 downregulated genes (fold-change > 2, *q* < 0.05, Fig. 4A), while 8 h RNA-seq only determined 30 downregulated and 16 upregulated genes (fold-change > 2, *q* < 0.05, Fig. S4A). Kyoto Encyclopedia of Genes and Genomes (KEGG) pathway analysis illustrated that downregulated cell cycle and up-regulated apoptosis pathways were enriched within SB743921-treated CCLP1 cells, which is consistent with the biological function of KSP inhibition (Fig. 4B). Considering KSP is crucial for centrosome separation and bipolar spindle assembly in the early stages of mitosis [21, 22], we examined the effect of KSP inhibition on the cell cycle process. Cell cycle analysis revealed a significant G2/M phase arrest in CCA cell lines treated with SB743921 (0.25 μM, 24 h), as well as other conventional mitotic inhibitors, supporting their inhibitory function on mitotic processes (Fig. 4C and Fig. S4C, D). In addition, treatment with SB743921 also increased cells in the Sub-G1 phase, representing dead cells, which revealed that KSP inhibition could cause growth inhibition and cell death in CCA cells (Fig. 4C and Fig. S4C, D). We further performed immunofluorescence staining of α-Tubulin, p-Histone 3 (p-H3), and DAPI in CCLP1 and SK-CHA-1 cells, to examine the effect of SB743921 on spindle formation. The results showed bipolar spindles were correctly assembled in control cells, whereas SB743921-treated cells mainly formed abnormal monoastral spindles (Fig. 4D–F).

To explore the underlying mechanism of SB743921-induced cell death, CCLP1 cells were treated with SB743921 alone or in combination with apoptosis inhibitor Z-VAD-FMK (Z-VAD, 50 μM), ferroptosis inhibitor Ferrostatin-1 (Ferro, 20 μM), necrosis inhibitor Necrostatin-1 (Nec-1, 20 μM) or ROS inhibitor N-Acetyl cysteine (Nac, 1 mM). A significant increment in the cell viability was observed after combined treatment of SB743921 with apoptosis inhibitor Z-VAD or ROS inhibitor Nac, not with Ferro or Nec-1, compared with a single treatment with SB743921, indicating that SB743921 mainly functioned through the apoptotic pathway (Fig. 4G). Besides, we performed the RealTime-Glo Annexin V Apoptosis and Necrosis assay, which showed that significant apoptosis signals appeared after 8 h of treatment with SB743921 (100 nM) in CCLP1 cells, much earlier than necrosis signals that emerged after 32 h following SB743921 treatment (Fig. 4H). Together, KSP inhibition was further confirmed to induce cell death mainly through the apoptosis process.

### Tumor suppression in CCA CDX models by KSP inhibition

**Fig. 5:**
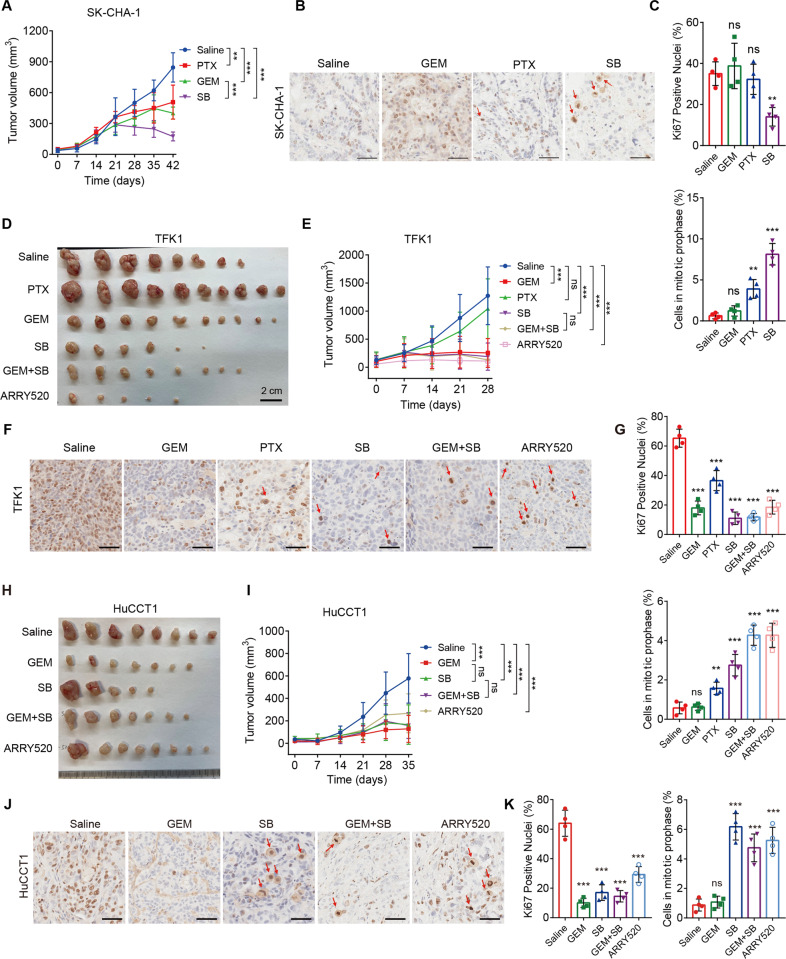
KSP inhibition caused tumor suppression in CCA CDX models. **A** Tumor volumes of SK-CHA-1 CDX models. Mice were allocated to four groups treated with SB743921 (5 mg/kg, *n* = 6), paclitaxel (10 mg/kg, *n* = 6), gemcitabine (10 mg/kg, *n* = 6), or saline (*n* = 6) twice a week for 6 weeks. **B**, **C** Representative images and quantification of Ki67 staining in SK-CHA-1 CDX tumors. Arrows indicate mitotic cells and the quantification of mitotic cells was shown. **D** Gross images of TFK1 CDX tumors. Mice were randomly allocated to different groups and treated with paclitaxel (10 mg/kg, *n* = 10), gemcitabine (10 mg/kg, *n* = 10), SB743921 (5 mg/kg, *n* = 6), ARRY520 (5 mg/kg, *n* = 5), gemcitabine plus SB743921 (*n* = 8), or saline (*n* = 8). **E** Tumor volumes of TFK1 CDX models. **F**, **G** Representative images and quantification of Ki67 staining in TFK1 CDX tumors. Arrows indicate mitotic cells and the quantification of mitotic cells was shown. **H** Gross images of HuCCT1 CDX tumors. Mice were randomly allocated to different groups and treated with gemcitabine (10 mg/kg, *n* = 7), SB743921 (5 mg/kg, *n* = 5), ARRY520 (5 mg/kg, *n* = 8), gemcitabine plus SB743921 (*n* = 7), or saline (*n* = 8). **I** Tumor volumes of HuCCT1 CDX models. **J**, **K** Representative images and quantification of Ki67 staining in HuCCT1 CDX tumors. Arrows indicate mitotic cells and the quantification of mitotic cells was shown. Scale bars, 50 μm. Panels **A**, **E**, and **I** were analyzed by two-way ANOVA; Panels **C**, **G**, and **K** were analyzed by the two-tailed unpaired *t-*test. **p < 0.01, ***p < 0.001, ns, not significant.

To investigate the therapeutic value of KSP inhibitors in vivo, CCA cell lines were subcutaneously injected into nude mice to establish cell line-derived xenograft (CDX) models. Mice were randomly allocated to control or treatment groups. In SK-CHA-1 CDX models, mice were intraperitoneally treated with SB743921 (5 mg/kg), paclitaxel (10 mg/kg), gemcitabine (5 mg/kg), or saline twice a week for six weeks. Mice were monitored for tumor size once a week. We found that treatment with SB743921 significantly decreased tumor growth, compared with saline treatment (*p* = 0.0198, after eight treatment injections, two-sided unpaired *t* test) (Fig. 5A). SB743921 showed more effectiveness on tumor inhibition, compared with paclitaxel (*p* = 0.0179, after eight treatment injections, two-sided unpaired *t* test) and gemcitabine monotherapy (*p* = 0.0232, after eight treatment injections, two-sided unpaired *t* test) (Fig. 5A). IHC staining showed tumors from paclitaxel or gemcitabine monotherapy cohorts had no difference in the number of proliferative (Ki67^+^) cells, compared with control tumors; whereas tumors from SB743921 cohorts displayed a significant decrease of Ki67^+^ cells (*p* = 0.0012, Fig. 5B, C). Moreover, SB743921 significantly increased a subpopulation of mitotic Ki67^+^ cells, identified by the condensed chromosomes (*p* < 0.001), suggesting SB743921 caused cell cycle arrest in the prophase of mitosis in vivo (Fig. 5C).

Considering standard chemotherapy for CCA is gemcitabine-based combined chemotherapy, we set up a group of gemcitabine plus SB743921 in consequent CDX treatment. Moreover, another KSP inhibitor ARRY520 was recruited to evaluate different KSP inhibition treatment strategies. TFK1 CDX models were established and mice were intraperitoneally treated with paclitaxel (10 mg/kg), gemcitabine (10 mg/kg), SB743921 (5 mg/kg), ARRY520 (5 mg/kg), gemcitabine (10 mg/kg) plus SB743921 (5 mg/kg), or saline separately twice a week for four weeks (Fig. 5D, E). TFK1 CDX tumors were markedly decreased after ARRY520 treatment (*p* < 0.001, after six treatment injections, two-sided unpaired *t* test). Both SB743921 and gemcitabine monotherapy showed significant tumor repression in TFK1 CDX models, compared with saline treatment (*p* = 0.002 for both groups, after six treatment injections, two-sided unpaired *t* test); whereas paclitaxel monotherapy had no impact on tumor growth. No synergistic effects on tumor suppression were observed in the cohort treated with gemcitabine plus SB743921 (Fig. 5E). In addition, tumors treated with KSP inhibitors SB743921 or ARRY520 showed significant decrease in the number of Ki67^+^ cells (Fig. 5F, G). Likewise, KSP inhibitors increased the population of chromosome-condensed Ki67^+^ cells, which confirmed the mitotic arrest-inducing effects of KSP inhibitors in vivo (Fig. 5F, G). Consistent with previous findings, KSP inhibitors SB743921 and ARRY520 also decreased tumor growth in HuCCT1 CDX models (*p* < 0.001 or *p* = 0.004, after eight treatment injections, two-sided unpaired *t* test). The combination of gemcitabine plus SB743921 had no synergistic effect in the treatment of HuCCT1 CDX tumors (Fig. 5H, I). Similarly, the KSP inhibitors SB743921 or ARRY520 treatment significantly decreased the number of Ki67^+^ cells, while increased the mitotically arrested cells (Fig. 5J, K). Moreover, cell apoptosis assessed by c-Caspase3 (c-Cas3) and TUNEL staining also showed significant increases in KSP inhibitor-treated CDX tumor samples (Fig. S5A–C), which indicated KSP inhibition had potent cytotoxicity to cause regression of CCA tumors in vivo.

Subsequently, the intratumoral uptake of SB743921 and PTX was quantified using the LC-MS/MS-based method. In TFK1-derived models, mice were intraperitoneally treated with SB743921 (5 mg/kg) or PTX (10 mg/kg), with tumors collected one hour later and sent for detection. The intratumoral content of SB743921 and PTX in TFK1-derived models was respectively depicted in Fig. S5D, revealing that the intratumoral uptake of SB743921 (14.99 ± 6.83 ng/g) was approximately six times higher than that in PTX (2.37 ± 0.68 ng/g). Besides, all mice tolerated well without significant weight loss and survived to the end of the experiments, demonstrating that the drug dosages used in our study were within safe limits (Fig. S5E). KSP inhibitors were reported to exhibit a maximum tolerated dose of milligram per kilogram in mice [31]. A phase I study of SB743921 in humans suggested the maximum-tolerated dose for SB743921 as a 1 h-infusion every 21 days is 4 mg/m^2^ [32]. The major dose-limiting toxicity of SB743921 was neutropenia. Together, higher in vivo bioavailability, tolerable safety, and efficacy indicated that KSP inhibitors are compounds with properties consistent with clinical application.

### Antitumor effects of SB743921 treatment in CCA PDX models

**Fig. 6:**
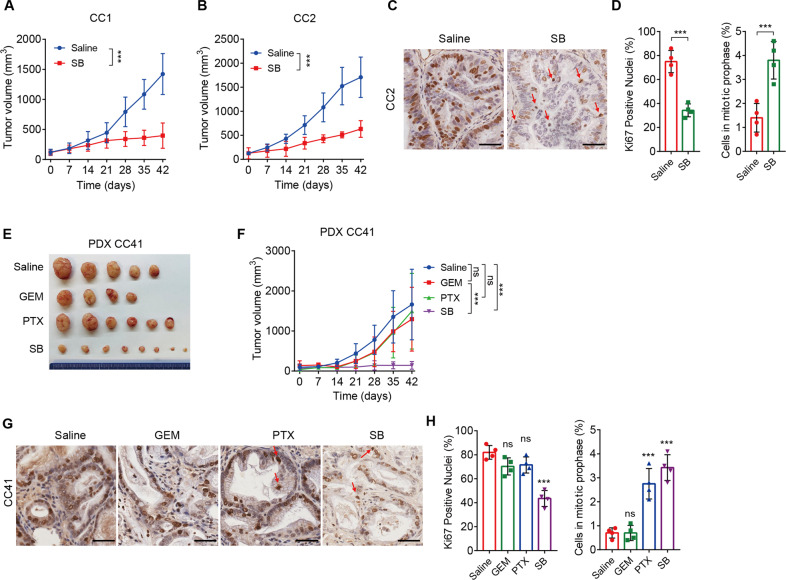
Tumor suppression resulting from SB743921 treatment in CCA PDX models. **A**, **B** Tumor volumes of CC1 and CC2 PDX models. PDX tumor-bearing mice were divided into two groups and treated with SB743921 (5 mg/kg, *n* = 4 and 3, respectively), or saline (*n* = 5 and 3 respectively). **C**, **D** Representative images and quantification of Ki67 staining in CC2 PDX tumors. Arrows indicate mitotic cells and the quantification of mitotic cells was shown. **E** Gross images of CC41 PDX tumors. **F** Tumor volumes of CC41 PDX models. PDX tumor-bearing mice were divided into four groups and treated with gemcitabine (10 mg/kg, *n* = 4), paclitaxel (10 mg/kg, *n* = 6), SB743921 (5 mg/kg, *n* = 8), or saline (*n* = 5). **G**, **H** Representative images and quantification of Ki67 staining in CC41 PDX tumors. Arrows indicate mitotic cells and the quantification of mitotic cells was shown. Scale bars, 50 μm. Panels **A**, **B**, and **F** were analyzed by two-way ANOVA; Panels **D** and **H** were analyzed by the two-tailed unpaired *t-*test. **p < 0.01, ***p < 0.001, ns, not significant.

We also tested the therapeutic response to SB743921 in CCA PDX models. Patient-derived xenograft (PDX) models are believed to preserve original tumor characteristics, including tumor heterogeneity, molecular signature, malignant phenotypes and genotypes, and tumor architecture, making them particularly suitable for evaluating the efficacy of novel tumor therapies [33, 34]. We have successfully established a series of well-characterized CCA PDX models as previously described [19]. Consistent with the CDX models, SB743921 treatment significantly decreased PDX tumor volumes in CC1 and CC2 PDX models (Fig. 6A, B). Likewise, SB743921-treated PDX tumors displayed a significant decrease of Ki67^+^ cells and an increase of mitotically arrested cells, compared with saline groups (Fig. 6C, D). However, treatment with gemcitabine or paclitaxel alone had limited therapeutic value in CC41 PDX tumors (Fig. 6E, F). Similarly, treatment with SB743921 decreased the Ki67^+^ cells but increased the mitotically arrested cells (Fig. 6G, H). Together, these findings demonstrated that KSP inhibition has the potential to decrease tumor growth of CCA in vivo.

## Discussion

CCA represents a substantial area of unmet medical need globally. Patients with advanced or metastatic CCA have an extremely unsatisfactory prognosis, mainly owing to limited treatment options and poor chemosensitivity. Novel and effective therapeutic approaches against CCA are urgently needed. Approximately 50% of CCA have at least one genetic mutation, and several targeted therapies have been developed in clinical trials. However, the effectiveness of targeted regimens in CCA patients is hampered by primary treatment resistance and short response durability [13]. Substantial heterogeneity of CCA at the genomic, epigenetic, and molecular levels severely compromises the efficacy of targeted therapies, which prompts us to envisage that strategies targeting common mechanisms of CCA may be valuable for complete tumor eradication and preventing the emergence of acquired resistance. Moreover, new broader therapeutic strategies are more urgent for patients who lack targetable molecular alterations.

Here, we performed a high-throughput drug screen in CCA cells using a library of approved or emerging antitumor compounds, thus identifying several drugs that were effective against CCA. In addition to some drugs previously proven to be available in CCA patients or preclinical models (such as gemcitabine, paclitaxel, 5-FU, dabrafenib, and bortezomib), we also found regimens not extensively investigated in CCA, among which mitotic inhibitors were within the top effective agents, including 3 traditional microtubule inhibitors and one novel alternative, KSP inhibitor SB743921. We selected these kinds of drugs as possibly effective broader therapeutic regimens for further study.

KSP functions as a motor protein that binds to microtubules in the early stages of mitosis [20–22]. It is responsible for centrosome separation and bipolar spindle assembly, which are essential for the proper segregation of chromosomes. Inhibition of KSP causes cell cycle arrest in mitosis with the formation of monopolar spindles, without any effect on non-proliferating cells. Various KSP inhibitors have progressed to clinical trials for the treatment of hematological malignancies and solid tumors such as hepatocellular carcinoma [23–26]. SB743921, as a second-generation KSP inhibitor, has shown antitumor activities in several types of tumors [23, 35, 36]. A phase I study of SB743921 has reported that one patient with CCA achieved partial response after 7 months from an initial treatment and remained until disease progression after nearly 12 months [32].

Consistent with previous findings in other types of tumors, we found that *KIF11* expression was highly expressed and associated with a dismal prognosis in CCA. CCA showed a higher *KIF11* gene dependency across various tumor types, suggesting the potential therapeutic intervention of targeting KSP. Indeed, SB743921 had significant cytotoxic activity in CCA cells, resulting in decreased growth both in CCA CDX and PDX tumors. KSP inhibitor ARRY520 also showed therapeutic effects on CCA CDX tumors, indicating a therapeutic role for KSP inhibition in CCA. Nevertheless, there is a possibility that the antitumor activity of KSP inhibitors in CCAs may be due to mechanisms other than KSP inhibition.

Given the complexity of oncogenic mechanisms responsible for CCA development and progression, combinatorial therapies will be necessary to improve outcomes and avoid acquired resistance in these patients. Paclitaxel-resistant cell lines were confirmed susceptible to SB743921 in vitro studies [32]. SB743921 has also been proven to overcome TKI resistance in chronic myeloid leukemia cells [36], suggesting KSP inhibition may act as an effective combination therapy for improving chemosensitivity and overcoming chemoresistance. Although our findings showed that a combination of SB743921 and gemcitabine had no synergistic effect on CDX tumors, the combination of KSP inhibitors with targeted therapies or other regimens may be a possibly promising therapeutic strategy, which requires further investigation. Further studies are also warranted to elucidate the function of KSP inhibitors in CCA, which will benefit for better understanding and application of this therapeutic strategy.

In addition, our study has potential limitations in terms of clinical translation, as with all preclinical drug-testing studies. The clinical efficacy of KSP inhibitors is attributed to the induction of mitotic arrest. Frequent cell division is thought to be a hallmark of tumor cells, however, this conception is mainly based on the rapid division rate of tumor cells observed in vitro or xenograft models, which are much faster than tumor cells in patient tumors [37]. Besides, the pharmacokinetic profile of KSP inhibitors in xenograft models may be different from human patients, and whether patients obtain adequate drug concentrations to kill tumor cells remains unknown. Therefore, the efficacy of mitotic inhibitors in patients may differ from preclinical studies.

In summary, we identified several compound candidates against CCA using a high-throughput drug screen, among which KSP inhibitor SB743921 showed significant therapeutic effects in CCA CDX and PDX models in vivo. Mechanistically, KSP inhibitors resulted in mitotic arrest with monopolar spindles in CCA cells. Moreover, our clinical data as well as the TCGA analysis showed *KIF11* expression was associated with poor outcomes in multiple tumors, indicating that this therapeutic regimen is worthy of further clinical investigation.

## Materials and methods

### In vitro cell culture

Human CCA cell lines (HCCC-9810, RBE, CCLP1, TFK1, SK-CHA-1, MZ-CHA-1, and CC143476) were used for in vitro studies. HCCC-9810, RBE, and CCLP1 cells were obtained from the Shanghai Cell Bank of the Chinese Academy of Sciences, China; TFK1, HuCCT1, and HIBEpiC cells were provided by S.-Q. Zou, Tongji Hospital, Huazhong University of Science and Technology; SK-CHA-1 and MZ-CHA-1 cell lines were offered by H. You, Xiamen University; CC143476 cells are primary cholangiocarcinoma cells isolated from human CCA tissues. All CCA cell lines were cultured in RPMI 1640 medium supplemented with 10% FBS, penicillin (100 IU/mL, Gibco), and streptomycin (100 μg/mL, Gibco). All cell lines were authenticated by DNA (short tandem repeat) profiling and tested for mycoplasma contamination.

### High-throughput drug screen and drug selection

A high-throughput drug screen was performed using PerkinElmer’s integrated robotic “explorer G3 workstations”. A library of 155 FDA-approved or emerging compounds was used in our study. Drugs were diluted at four indicated concentrations and dispensed into 96-well plates according to the sequence shown in Fig. 1A. CCA cell lines were seeded into 384-well plates, 3000 cells per well for a total volume of 20 μL. After 24 h, drugs were added to each well and incubated for 72 h. Cell viability was subsequently measured using the CellTiter-Glo luminescent cell viability assay (G7571, Promega) according to the manufacturer’s instructions. Luminescence was measured using a Synergy2 plate reader and the Gen5 software (BioTek). The raw data were analyzed using the Breeze pipeline (https://breeze.fimm.fi) for curve fitting and to obtain quality control and drug response parameters including Z-factor, AUC, and DSS as previously described [23]. The AUC is defined as the area under the dose-response curve, compared to the total graph area, above a 10% threshold. The response of different drugs was compared using the DSS (drug sensitivity score), where a DSS ≥ 10 was considered an effective response. The IC_50_ was also used to select drugs with activity at low drug concentrations (IC_50_ < 1000 nM).

### *KIF11* knockdown

*KIF11* siRNA oligonucleotides were designed and synthesized by GenePharma (Shanghai, China). The siRNA sequences were as follows: siKIF11 #1, 5′-GCCUGAAGUGAAUCAGAAACU-3′; siKIF11 #2, 5’-UGAUAUAUUCUUCUUUAACAA-3′; Lipo8000 (C0533, Beyotime) was used to transfect these siRNAs into CCA cells, according to the manufacturer’s standard protocols.

### Cell survival, colony formation, and viability assay

For cell survival assay, cells were seeded at a concentration of 3000–5000 cells per well in 96-well plates and treated with indicated doses of drugs for 72 h. Subsequently, cells were tested using the CellTiter-Glo luminescent cell viability assay. For colony formation assay, equivalent amounts of cells were plated in 6-well plates at a density of 5000 cells/well and treated with indicated doses of drugs 36 h later. After another one-two weeks, colonies were stained with crystal violet and then counted for quantification. For cell viability assessment, cells were seeded and cultured in 6-well plates and treated with indicated doses of drugs for 24 h. Cells were incubated with PI (P4170; Sigma-Aldrich) for 15 min, washed, resuspended in PBS, and then observed by fluorescence microscopy (Leica Biosystems). All experiments were performed in triplicate. All drugs used in the study, including KSP inhibitor SB743921, vincristine, paclitaxel, docetaxel, cabazitaxel, cephalomannine, homoharringtonine, and gemcitabine, were purchased from Selleck.

### Real-time monitoring of cell apoptosis and necrosis

The RealTime-Glo Annexin V Apoptosis and Necrosis Assay (Promega) was used for the real-time monitoring of apoptosis and necrosis signals, according to the manufacturer’s standard protocols. Luminescence and fluorescence in a 96-well white bottom-permeable plate were measured every 8 h using a microplate reader (Synergy H1, BioTek). Each treatment was performed in triplicate. The luminescence signal indicates apoptosis and the fluorescence signal indicates necrosis. After 48 h of continuous monitoring, apoptosis and necrosis signal curves were plotted using GraphPad software (version 7.0).

### Cell cycle analysis

Cells were treated with indicated doses of drugs for 24 h, and then fixed in ice-cold 70% ethanol and kept at −20 °C. Upon analysis, they were washed in 1 × phosphate-buffered saline and incubated on ice for 30 min in Vindelöv solution (3.5 μM Tris-HCl (pH 7.6), 10 mM NaCl, PI (50 μg/mL), ribonuclease (20 μg/mL), and 0.1% (v/v) NP-40). Samples were run on a FACSVerse flow cytometer (BD Biosciences), and data were analyzed using FlowJo software. All experiments were performed in triplicate.

### Bioinformatics analyses

UALCAN (http://ualcan.path.uab.edu/analysis.html) is a comprehensive web resource that provides analyses based on The Cancer Genome Atlas (TCGA) data. Expression data for *KIF11* was obtained using the “Expression Analysis” module of UALCAN and the “KIRC” dataset. *P* value was generated using Student’s *t*-test. The *p* value cutoff was 0.05.

HPA (https://www.proteinatlas.org/ENSG00000138160-KIF11/pathology) is an analysis tool containing numerous catalogs of protein-coding genes with detailed expression information. We performed a differential mRNA expression analysis of tumors and normal tissues, pathological stage analysis, and correlative prognostic analysis of *KIF11* in our study. *P* value was generated using Student’s *t*-test in expression and pathological stage analysis. The *p* value cutoff was 0.05. Prognostic analysis was performed using a Kaplan–Meier curve.

Dependency scores were obtained from the DepMap portal (https://depmap.org/portal/) at the Broad Institute by downloading the CRISPR dataset (DepMap Public 19Q3, dataset 10.6084/m9.figshare.9201770.v1.). A lower CERES score indicates a likelihood that the gene of interest is essential in the given cell line. A score of 0 indicates a gene is not essential; correspondingly, −1 is comparable to the median of all pan-essential genes [29].

### Immunoblot

Cells were collected and lysed in radioimmunoprecipitation assay buffer (Beyotime; 50 mM tris-HCl (pH 7.4), 1% NP-40, 0.5% Na-deoxycholate, 0.1% SDS, 150 mM NaCl, 2 mM EDTA, and 50 mM NaF) including a cocktail of protease (Topscience; 1 mM AEBSF.HCl, 1 μM aprotinin, 50 μM bestatin, 15 μM E-64, 20 M leupeptin, 15 μM pepstatin A) and phosphatase (Topscience; 1 mM sodium fluoride, 1 mM sodium orthovanadate, 1.15 mM sodium molybdate, 4 mM sodium tartrate, 2 mM imidazole, 25 M (−)-p-bromotetramisole oxalate, 5 μM cantharidin, 5 nM microcystin-LR) inhibitors. Immunoblots were performed following the standard protocol and detected using an Odyssey fluorescence scanner (LI-COR). All experiments were performed in triplicate. Antibodies used are *KIF11*/KSP/Eg5 (1:1000; #23333-1-AP, Proteintech), p53 (1:1000; #10442-1-AP, Proteintech), c-Caspase3 (1:1000; #19677-1-AP, Proteintech), GAPDH (1:1000; #10494-1-AP, Proteintech), p-CHK1 (1:1000, #12302, CST), p-P53 (1:1000, #2521, CST), γ-H2A.x (1:1000; #9718, CST), PARP (1:1000; #9532, CST).

### Immunohistochemistry (IHC) and Immunofluorescence (IF)

For IHC, tumor tissues were fixed in 10% formalin overnight and embedded in paraffin. The tumors were cut in 4 μm sections and stained. The endogenous peroxidases were inactivated by 3% hydrogen peroxide. Nonspecific signals were blocked using 1% bovine serum albumin. Tissue slides were incubated with the primary antibody: KSP/Eg5/*KIF11* (1:100, #23333-1-AP, Proteintech), Ki67 (1:400, #ab15580, Abcam) overnight at 4 °C, washed for three times using PBS buffer and subsequently incubated with secondary antibody (HRP-Polymer, Biocare Medical) for 30 min at room temperature. The slides were then stained with 3,3′-diaminobenzidine (DAB) substrate (Thermo Fisher Scientific), and counterstained with hematoxylin, and mounted with a mounting medium. Tissue sections were reviewed and scored blindly for staining intensity (0–3) by two independent pathologists using the Aperio ImageScope Viewer. High expression of *KIF11* corresponded to a staining score of 2 or 3, whereas low expression corresponded to staining scores of 0 and 1. For IF, CCLP1 and SK-CHA-1 cells were treated with SB743921 or DMSO for 24 h. Cells were fixed in 4% paraformaldehyde, permeabilized with 0.2% Triton X-100, and blocked using 10% goat serum. Staining of microtubules and centrosomes was performed using a p-Histone 3 antibody (1:100; #53348, CST) and α-tubulin antibody (1:100; #ab7291, Abcam). Secondary fluorescent antibodies were purchased from Invitrogen. Cell nuclei were visualized with 4′,6-diamidino-2-phenylindole (DAPI) staining (1:1000; #D3571, Invitrogen).

### RNA sequencing

CCLP1 cells were treated with SB743921 or vehicle control for 8 and 16 h. RNA extraction was performed following the standard protocol, and 500 ng of total RNA was used for input to library preparation. For RNA-seq analysis, 2 × 150 bp paired-end sequencing (PE150) was generated on an Illumina Novaseq 6000 (LC-Bio Technology CO., Ltd.). The fastp software (https://github.com/OpenGene/fastp) was used to remove the reads that contained adaptor contamination, low-quality bases, and undetermined bases with default parameters. HISAT2 (https://ccb.jhu.edu/software/hisat2) was used to map reads to the reference genome of Homo sapiens GRCh38. StringTie was used to perform expression levels for mRNAs by calculating FPKM (fragments per kilobase of exon model per million mapped fragments). Genes with FPKM <1 in all samples were excluded in subsequent analyses. For the remaining genes, all FPKM values less than 1 were set to 1. The differentially expressed mRNAs were selected with fold change > 2 and with parametric F-test comparing nested linear models (*q* < 0.05) by R package edgeR.

### Xenograft tumor experiments

Nude mice (female, 6w) were purchased from Shanghai Sippe-Bk Lab Animal Co.,Ltd. All procedures were conducted according to the guidelines from the Regional Ethics Committee for Animal Research (ethical permit no. M11-15 and 19012-19).

For CDX models, CCA cell lines (1 × 10^6^) were suspended in a 100 μL mixture of medium and Matrigel (2:1) and injected subcutaneously into the flanks of 6-week-old female nude mice. When tumors reached about 100 mm^3^, each mouse was randomly allocated to control or treatment groups. Mice were treated by intraperitoneal injection with SB743921 (5 mg/kg), paclitaxel (10 mg/kg), gemcitabine (10 mg/kg), ARRY520 (5 mg/kg), or saline separately, twice a week for indicated times (4–6 weeks). Tumor sizes were monitored using a caliper once a week, and tumor volumes were calculated using the 0.5 × L × W^2^ (L is the longest diameter and W is the shortest diameter). Mice were euthanized by CO_2_ asphyxiation three days after the last treatment and tumor tissues were collected for further study. PDX models were established and transplanted to the next generation as previously described [19]. Briefly, when PDX tumors of the second generation reached about 100 mm^3^, nude mice were randomly assigned to control or treatment groups. The subsequent procedures are the same with CDX models. Mice were monitored for weight loss and other signs of toxicity. Treatment was paused if mice displayed weight loss ≥ 10%. Mice were euthanized when they showed abnormal signs or symptoms due to tumor burden or at weight loss ≥ 15% of initial weight.

Sample size calculation was an important process to show the validity, accuracy, and reliability of a study. Some software and calculators are extremely helpful to calculate sample sizes, such as Power Analysis & Sample Size (PASS). Since our animal experiments are pilot and exploratory studies, it may be more appropriate to consider other things that can be tested while maintaining scientific and qualitative levels than estimating sample size. Besides, those studies are designed as blind experiments. The investigator who was in charge of measuring tumor volume and weight was blinded to the group allocation during the experiment.

### Detection of intratumoral paclitaxel and SB743921

Quantification of intratumoral paclitaxel and SB743921 was performed using the LC-MS/MS-based method, as previously described [38]. Separation was achieved by a 5 μL injection of the prepared dried cell extracts on a Kinetex 2.6 μm C18 100A column (Phenomenex) with a column temperature of 35 °C. A gradient elution utilizing 0.1% formic acid (FA) in water as solvent A and 0.1% FA in acetonitrile as solvent B was performed, with a stable flow rate of 0.5 mL/min. The gradient was as follows: 0 min (90% A and 10% B), 0.2 min (90% A and 10% B), 1.7 min (5% A and 95% B), 3 min (5% A and 95% B), 3.01 min (90% A and 10% B), and 4.5 min (90% A and 10% B). Mass spectrometry conditions: electrospray ionization (ESI) source, positive ionization mode. The ion source temperature (TEM) was 600 °C, ion spray voltage (IS) 5500 V, ion source gas 1 (GS1) 50 psi, ion source gas 2 (GS2) 60 psi, curtain gas (CUR) 25 psi. Scanning was performed by multiple reaction monitoring (MRM). Data analysis: The working standard solutions were separately analyzed using LC-MS. The concentration of the working standard solution was used as the horizontal coordinate and the peak area was used as the vertical coordinate to examine the linear range and plot the standard curve. All samples were quantitatively analyzed according to the established sample pre-treatment and instrumental analysis methods.

### Human CCA cohort

Human CCA specimens and patients’ clinical data were obtained from the Renji Hospital, Shanghai Jiao Tong University, Shanghai, with the approval of the Research Ethics Committee of Renji Hospital. Diagnoses of all patients were confirmed by pathological analyses. Informed consent was obtained from all patients. Inclusion criteria: (1) no history of other malignant tumors within 5 years before surgery; (2) no other history of anti-tumor therapy before surgery; (3) between 20 and 75 years old. Exclusion criteria: (1) palliative resection; (2) perioperative death; (3) incomplete clinicopathological data; (4) incomplete follow-up information. Patient characteristics including age, gender, tumor size, tumor number, and other tumor parameters relevant to the study are shown in Supplementary Data File 2.

### Statistical analyses

All grouped data were presented as box plots in figures. Statistical comparison between the data from different groups was performed in PRISM v.7 software (GraphPad) using either a two-tailed *t*-test or one-way analysis of variance (ANOVA) with post hoc analysis, as indicated in the figure legends. For tumor growth curves, *p* values were determined by two-way ANOVA. *p* < 0.05 was considered statistically significant, and the asterisked *p* values are indicated in the figures. Data were presented as mean ± SD, and sample sizes are indicated in the figure legends. For all experiments, more than three biological replicates under the same condition were performed. Representative results from at least two independent experiments were shown. For survival analyses, Kaplan–Meier survival curves were generated by SPSS 18.0, and the log-rank test was performed to assess the statistical significance of differences between the two groups.

## Supplementary information

Supplementary Figure 1

Supplementary Figure 2

Supplementary Figure 3

Supplementary Figure 4

Supplementary Figure 5

Supplementary Figure Legends

Supplementary Data file 1

Supplementary Data file 2

Original Data File

Original Data File

Checklist

## Data Availability

All data needed to evaluate the conclusions in the paper are presented in the paper and/or in the Supplementary Data files. RNA sequencing data is available at NCBI Gene Expression Omnibus (GEO) database (https://www.ncbi.nlm.nih.gov/geo/) under accession number GSE212232.

## References

[1] Banales JM, Cardinale V, Carpino G, Marzioni M, Andersen JB, Invernizzi P (2016). Expert consensus document: Cholangiocarcinoma: Current knowledge and future perspectives consensus statement from the European Network for the Study of Cholangiocarcinoma (ENS-CCA). Nat Rev Gastroenterol Hepatol.

[2] Rizvi S, Gores GJ (2013). Pathogenesis, diagnosis, and management of cholangiocarcinoma. Gastroenterology.

[3] Bertuccio P, Malvezzi M, Carioli G, Hashim D, Boffetta P, El-Serag HB (2019). Global trends in mortality from intrahepatic and extrahepatic cholangiocarcinoma. J Hepatol.

[4] Rizvi S, Khan SA, Hallemeier CL, Kelley RK, Gores GJ (2018). Cholangiocarcinoma—evolving concepts and therapeutic strategies. Nat Rev Clin Oncol.

[5] Saha SK, Zhu AX, Fuchs CS, Brooks GA (2016). Forty-year trends in cholangiocarcinoma incidence in the U.S.: Intrahepatic disease on the rise. Oncologist.

[6] Forner A, Vidili G, Rengo M, Bujanda L, Ponz-Sarvise M, Lamarca A (2019). Clinical presentation, diagnosis and staging of cholangiocarcinoma. Liver Int.

[7] Jarnagin WR, Fong Y, DeMatteo RP, Gonen M, Burke EC, Bodniewicz BJ (2001). Staging, resectability, and outcome in 225 patients with hilar cholangiocarcinoma. Ann Surg.

[8] Valle JW, Borbath I, Khan SA, Huguet F, Gruenberger T, Arnold D (2016). Biliary cancer: ESMO Clinical Practice Guidelines for diagnosis, treatment, and follow-up. Ann Oncol.

[9] Okusaka T, Nakachi K, Fukutomi A, Mizuno N, Ohkawa S, Funakoshi A (2010). Gemcitabine alone or in combination with cisplatin in patients with biliary tract cancer: A comparative multicentre study in Japan. Br J Cancer.

[10] Valle J, Wasan H, Palmer DH, Cunningham D, Anthoney A, Maraveyas A (2010). Cisplatin plus gemcitabine versus gemcitabine for biliary tract cancer. N Engl J Med.

[11] Shroff RT, Javle MM, Xiao L, Kaseb AO, Varadhachary GR, Wolff RA (2019). Gemcitabine, cisplatin, and nab-paclitaxel for the treatment of advanced biliary tract cancers: A phase 2 clinical trial. JAMA Oncol.

[12] Abou-Alfa GK, Macarulla T, Javle MM, Kelley RK, Lubner SJ, Adeva J (2020). Ivosidenib in IDH1-mutant, chemotherapy-refractory cholangiocarcinoma (ClarIDHy): A multicentre, randomised, double-blind, placebo-controlled, phase 3 study. Lancet Oncol.

[13] Abou-Alfa GK, Sahai V, Hollebecque A, Vaccaro G, Melisi D, Al-Rajabi R (2020). Pemigatinib for previously treated, locally advanced or metastatic cholangiocarcinoma: a multicentre, open-label, phase 2 study. Lancet Oncol.

[14] Javle M, Lowery M, Shroff RT, Weiss KH, Springfeld C, Borad MJ (2018). Phase II study of BGJ398 in patients with FGFR-altered advanced cholangiocarcinoma. J Clin Oncol.

[15] Atkinson JM, Shelat AA, Carcaboso AM, Kranenburg TA, Arnold LA, Boulos N (2011). An integrated in vitro and in vivo high-throughput screen identifies treatment leads for ependymoma. Cancer Cell.

[16] Hansson K, Radke K, Aaltonen K, Saarela J, Manas A, Sjolund J (2020). Therapeutic targeting of KSP in preclinical models of high-risk neuroblastoma. Sci Transl Med.

[17] Pei Y, Liu KW, Wang J, Garancher A, Tao R, Esparza LA (2016). HDAC and PI3K antagonists cooperate to inhibit growth of MYC-driven medulloblastoma. Cancer Cell.

[18] Manasanch EE, Orlowski RZ (2017). Proteasome inhibitors in cancer therapy. Nat Rev Clin Oncol.

[19] Jiang TY, Pan YF, Wan ZH, Lin YK, Zhu B, Yuan ZG, et al. PTEN status determines chemosensitivity to proteasome inhibition in cholangiocarcinoma. Sci Transl Med. 2020;12:eaay0152. 10.1126/scitranslmed.aay0152 32967970

[20] Blangy A, Lane HA, d’Herin P, Harper M, Kress M, Nigg EA (1995). Phosphorylation by p34cdc2 regulates spindle association of human Eg5, a kinesin-related motor essential for bipolar spindle formation in vivo. Cell.

[21] Hagan I, Yanagida M (1990). Novel potential mitotic motor protein encoded by the fission yeast cut7+ gene. Nature.

[22] Zhang Y, Xu W (2008). Progress on kinesin spindle protein inhibitors as anti-cancer agents. Anticancer Agents Med Chem.

[23] Bongero D, Paoluzzi L, Marchi E, Zullo KM, Neisa R, Mao Y (2015). The novel kinesin spindle protein (KSP) inhibitor SB-743921 exhibits marked activity in in vivo and in vitro models of aggressive large B-cell lymphoma. Leuk Lymphoma.

[24] Holen K, DiPaola R, Liu G, Tan AR, Wilding G, Hsu K (2012). A phase I trial of MK-0731, a kinesin spindle protein (KSP) inhibitor, in patients with solid tumors. Invest N Drugs.

[25] Khoury HJ, Garcia-Manero G, Borthakur G, Kadia T, Foudray MC, Arellano M (2012). A phase 1 dose-escalation study of ARRY-520, a kinesin spindle protein inhibitor, in patients with advanced myeloid leukemias. Cancer.

[26] LoRusso PM, Goncalves PH, Casetta L, Carter JA, Litwiler K, Roseberry D (2015). First-in-human phase 1 study of filanesib (ARRY-520), a kinesin spindle protein inhibitor, in patients with advanced solid tumors. Invest N Drugs.

[27] Yadav B, Pemovska T, Szwajda A, Kulesskiy E, Kontro M, Karjalainen R (2014). Quantitative scoring of differential drug sensitivity for individually optimized anticancer therapies. Sci Rep.

[28] Phelip JM, Edeline J, Blanc JF, Barbier E, Michel P, Bourgeois V (2019). Modified FOLFIRINOX versus CisGem first-line chemotherapy for locally advanced non resectable or metastatic biliary tract cancer (AMEBICA)-PRODIGE 38: Study protocol for a randomized controlled multicenter phase II/III study. Dig Liver Dis.

[29] Subbiah V, Lassen U, Elez E, Italiano A, Curigliano G, Javle M (2020). Dabrafenib plus trametinib in patients with BRAF(V600E)-mutated biliary tract cancer (ROAR): A phase 2, open-label, single-arm, multicentre basket trial. Lancet Oncol.

[30] Meyers RM, Bryan JG, McFarland JM, Weir BA, Sizemore AE, Xu H (2017). Computational correction of copy number effect improves specificity of CRISPR-Cas9 essentiality screens in cancer cells. Nat Genet.

[31] Woessner R, Tunquist B, Lemieux C, Chlipala E, Jackinsky S, Dewolf W (2009). ARRY-520, a novel KSP inhibitor with potent activity in hematological and taxane-resistant tumor models. Anticancer Res.

[32] Holen KD, Belani CP, Wilding G, Ramalingam S, Volkman JL, Ramanathan RK (2011). A first in human study of SB-743921, a kinesin spindle protein inhibitor, to determine pharmacokinetics, biologic effects and establish a recommended phase II dose. Cancer Chemother Pharm.

[33] Hidalgo M, Amant F, Biankin AV, Budinska E, Byrne AT, Caldas C (2014). Patient-derived xenograft models: An emerging platform for translational cancer research. Cancer Discov.

[34] Tentler JJ, Tan AC, Weekes CD, Jimeno A, Leong S, Pitts TM (2012). Patient-derived tumour xenografts as models for oncology drug development. Nat Rev Clin Oncol.

[35] Song IS, Jeong YJ, Nyamaa B, Jeong SH, Kim HK, Kim N (2015). KSP inhibitor SB743921 induces death of multiple myeloma cells via inhibition of the NF-kappaB signaling pathway. BMB Rep.

[36] Yin Y, Sun H, Xu J, Xiao F, Wang H, Yang Y (2015). Kinesin spindle protein inhibitor SB743921 induces mitotic arrest and apoptosis and overcomes imatinib resistance of chronic myeloid leukemia cells. Leuk Lymphoma.

[37] Salmela AL, Kallio MJ (2013). Mitosis as an anti-cancer drug target. Chromosoma.

[38] Halbrook CJ, Pontious C, Kovalenko I, Lapienyte L, Dreyer S, Lee HJ (2019). Macrophage-released pyrimidines inhibit gemcitabine therapy in pancreatic cancer. Cell Metab.

