# Supplementary figures and images for: The therapeutic effect of KSP inhibitors in preclinical models of cholangiocarcinoma
Source: Cell Death Dis. 2022 Sep 19;13(9):799. doi: 10.1038/s41419-022-05247-0 (PMC9485230; doi:10.1038/s41419-022-05247-0)

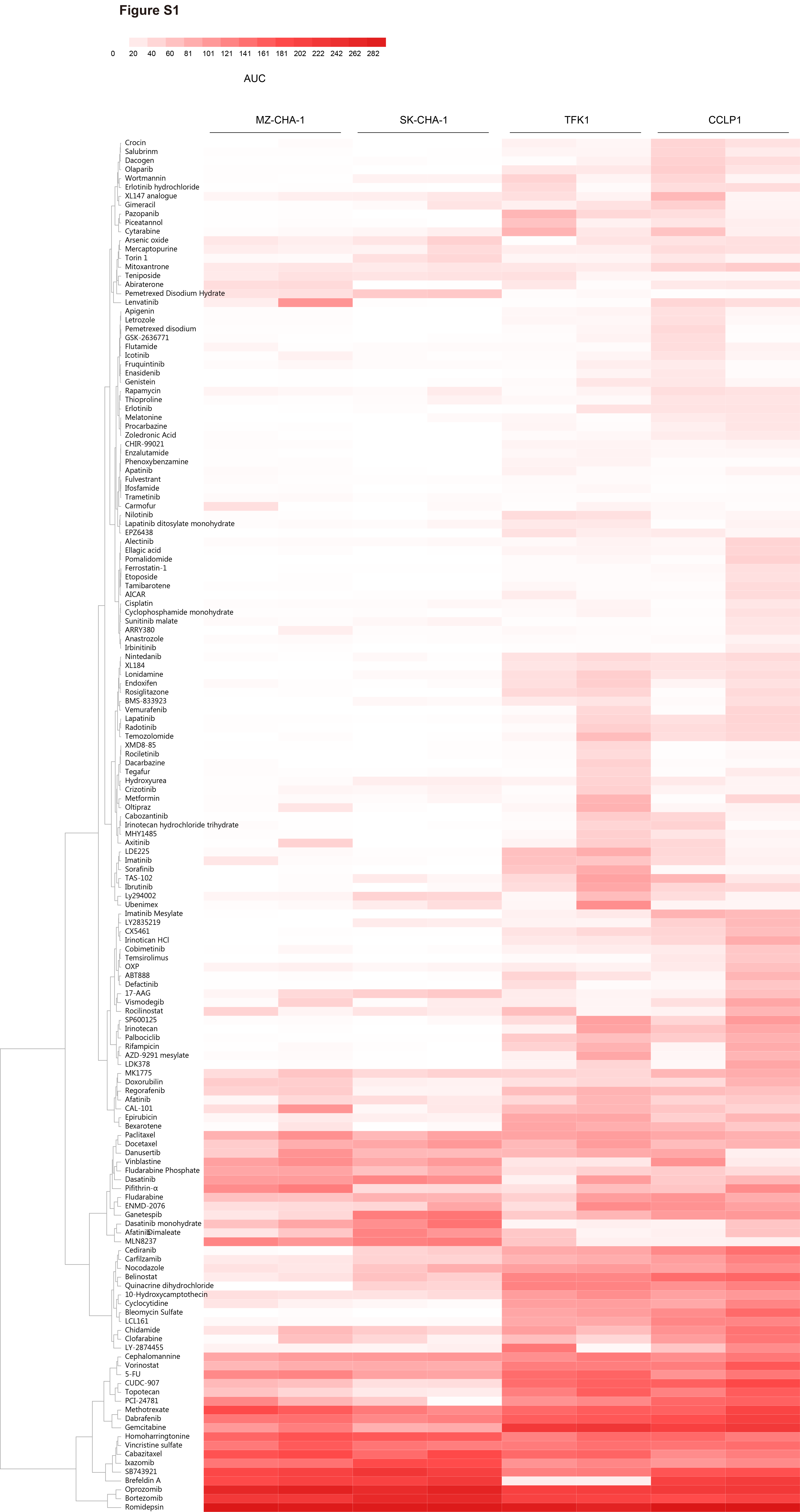

Supplement: Supplementary file 1 — Supplementary Figure 1 [file 41419_2022_5247_MOESM1_ESM.png]

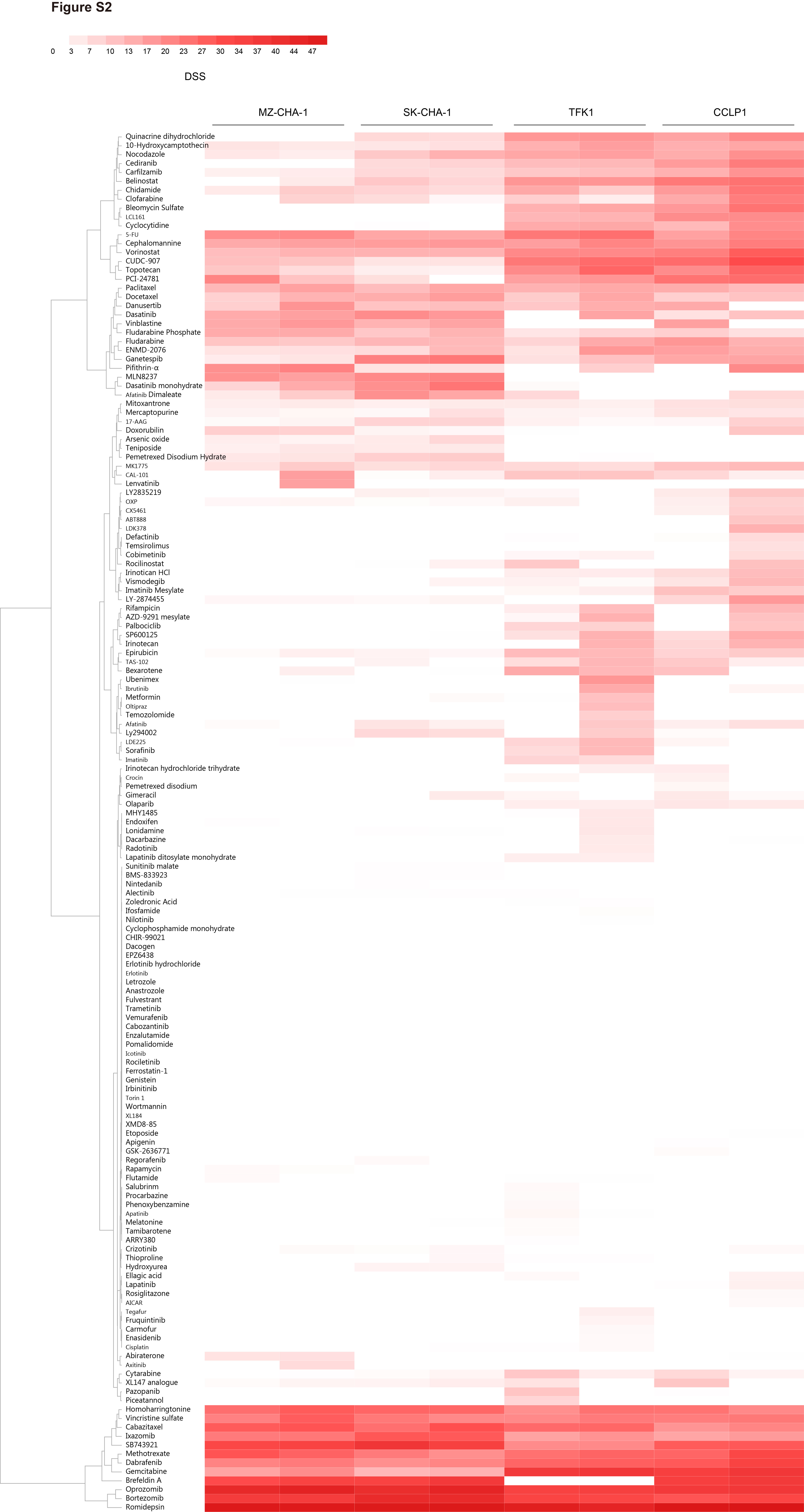

Supplement: Supplementary file 2 — Supplementary Figure 2 [file 41419_2022_5247_MOESM2_ESM.png]

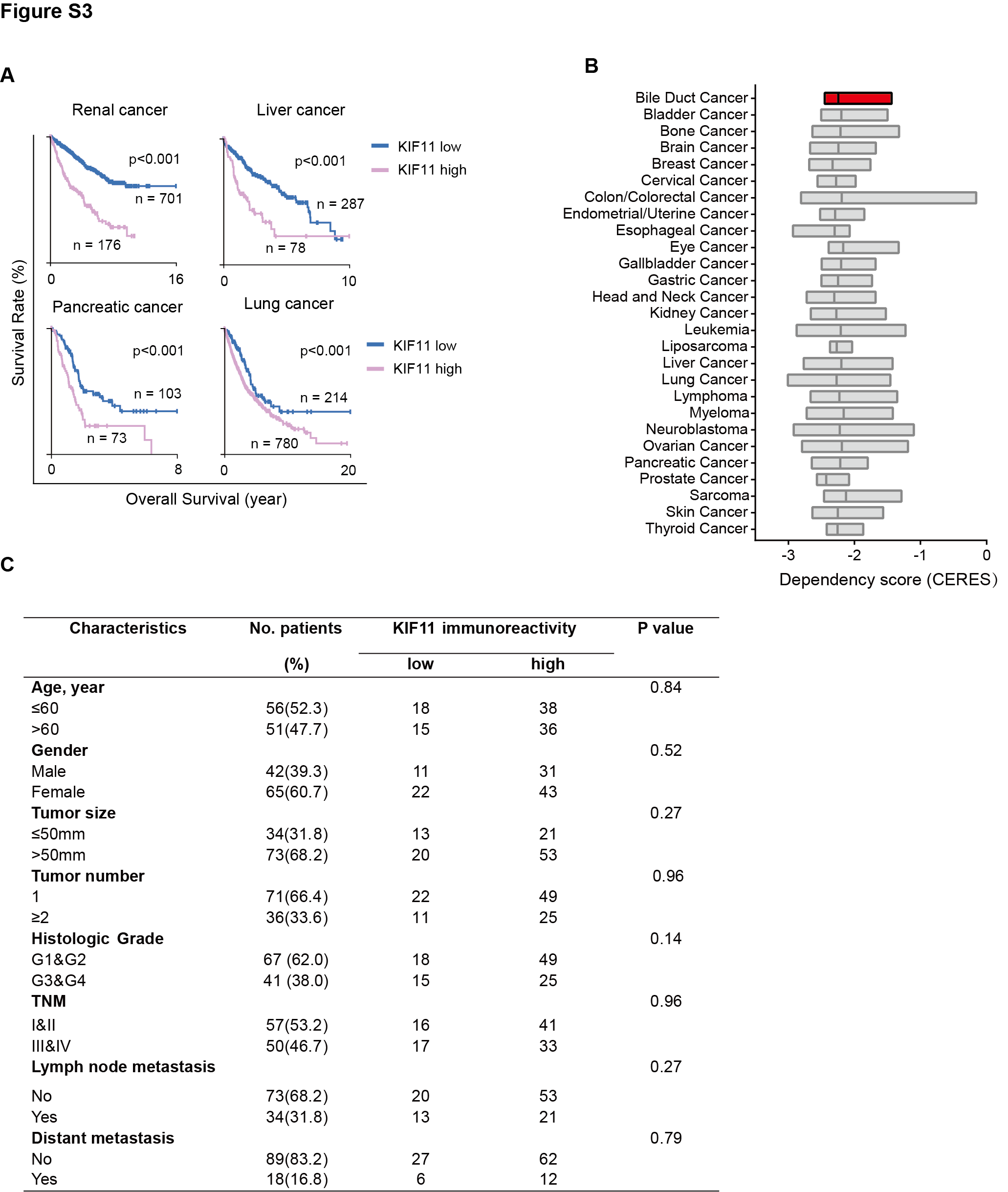

Supplement: Supplementary file 3 — Supplementary Figure 3 [file 41419_2022_5247_MOESM3_ESM.png]

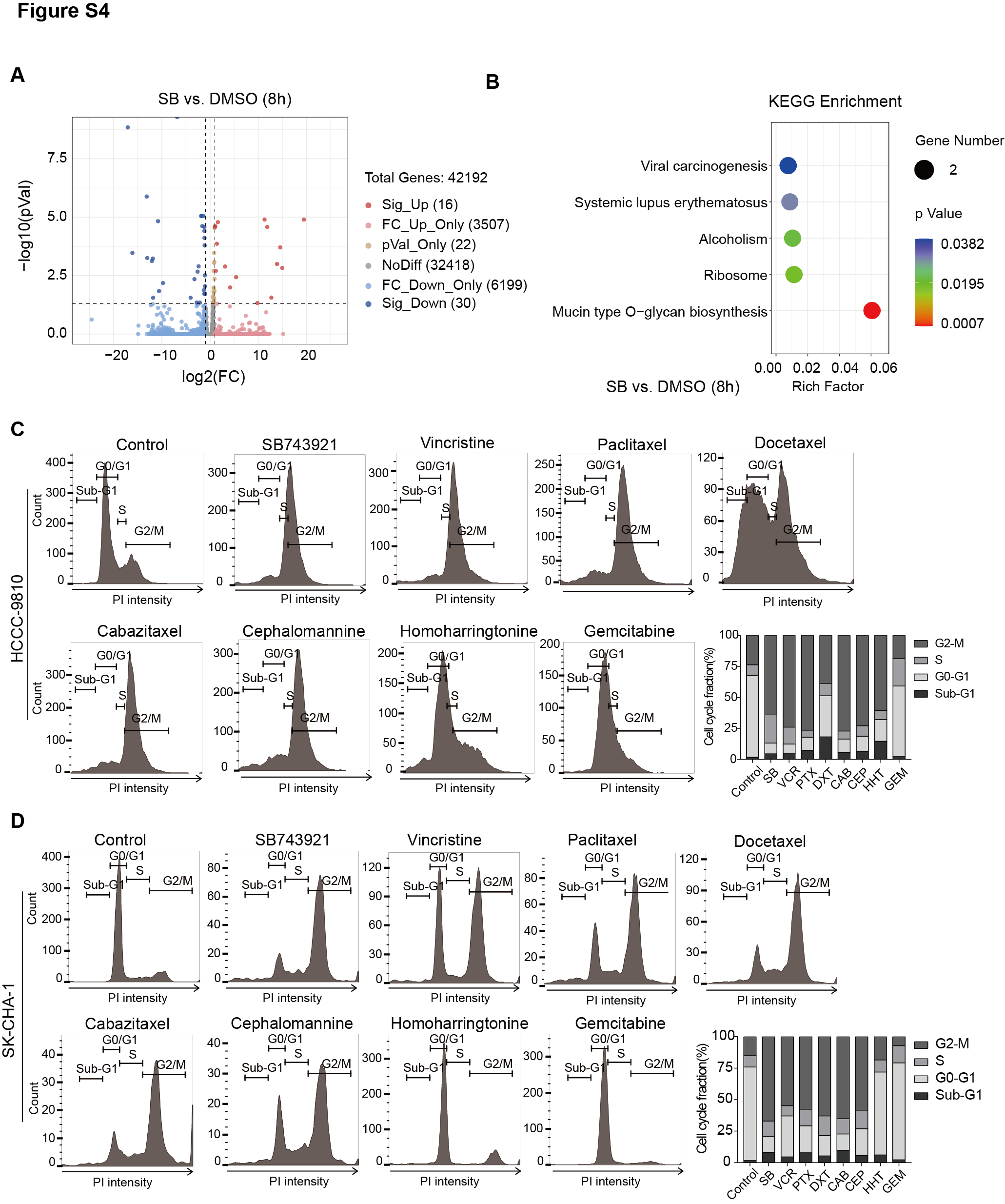

Supplement: Supplementary file 4 — Supplementary Figure 4 [file 41419_2022_5247_MOESM4_ESM.png]

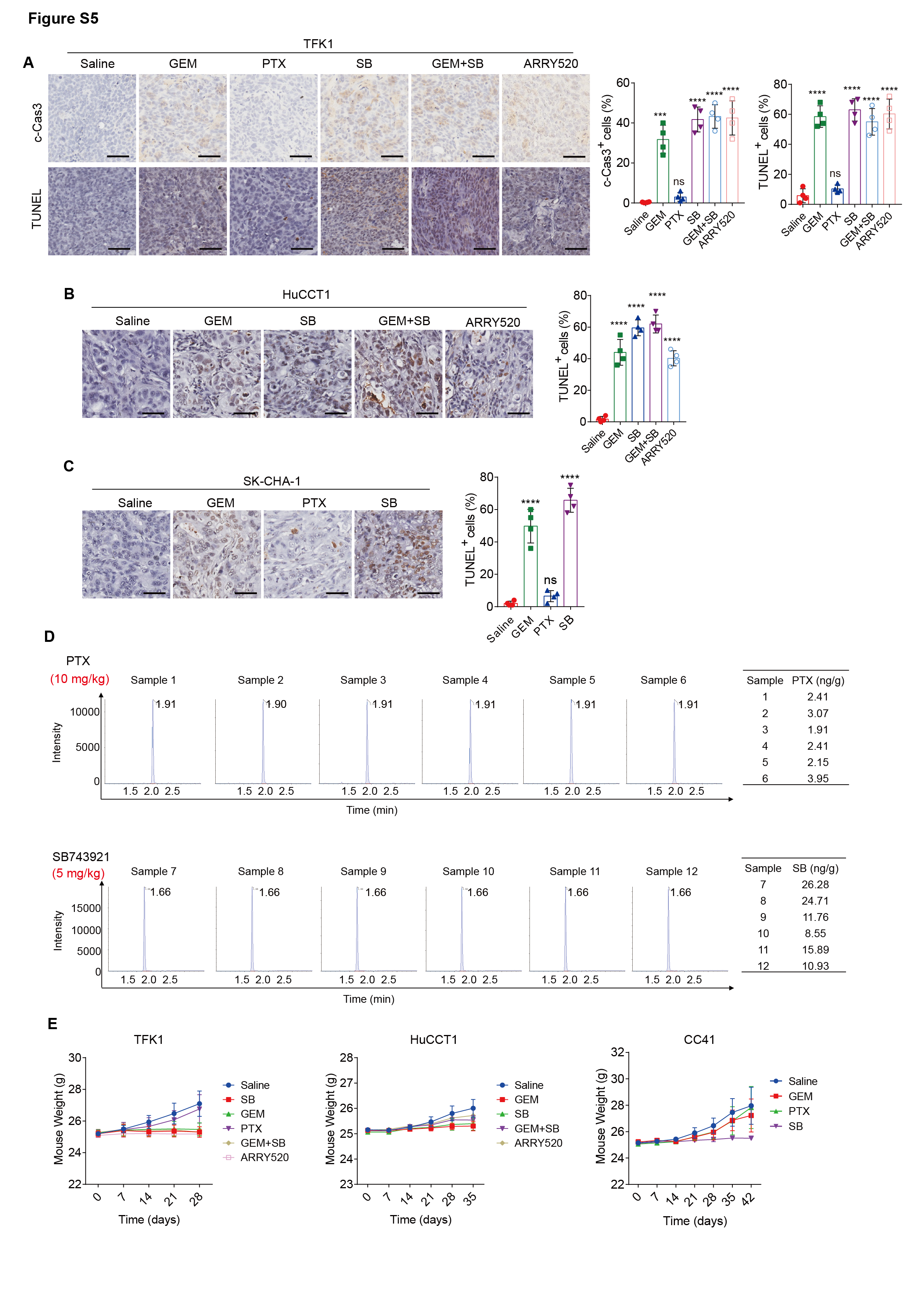

Supplement: Supplementary file 5 — Supplementary Figure 5 [file 41419_2022_5247_MOESM5_ESM.png]
