# Supplementary material for: The therapeutic effect of KSP inhibitors in preclinical models of cholangiocarcinoma
Source: Cell Death Dis. 2022 Sep 19;13(9):799. doi: 10.1038/s41419-022-05247-0 (PMC9485230; doi:10.1038/s41419-022-05247-0)
Supplement: Supplementary file 6 — Supplementary Figure Legends [file 41419_2022_5247_MOESM6_ESM.doc]

**Supplementary Figure Legends**

**Fig. S1. Heatmap of AUC for each drug in four CCA cells**.

AUC is the area covered between the dose-response curve and the concentration x-axis.

**Fig. S2. Heatmap of the DSS over all the 155 compounds in four CCA cells**.

Drugs with DSS ≥ 10 were regarded as sensitive drugs.

**Fig. S3. *KIF11* gene expression and dependency in pan-cancers.**

**(A)** Survival of patients with high and low *KIF11* gene expression in renal, liver, pancreatic, and lung cancers using Kaplan-Meier survival curves. Statistical analyses were performed using the log-rank test. **(B)** *KIF11* dependency across 27 tumor types was presented using the CERES dependency score. A lower score indicates higher gene dependency. **(C)** Relationship between *KIF11* expression and clinicopathologic characteristics in human CCA samples.

**Fig. S4. SB743921 causes cell cycle arrest of CCA.**

**(A)** Volcano plot of RNA-seq data from CCLP1 cells treated with SB743921 (10 nM, 8 h). Gene-expression alteration in the treated group was normalized by corresponding DMSO control. Significant genes were determined by Student’s t test and a threshold cutoff of q < 0.05, 2-fold change. Red, induced; green, repressed. **(B)** KEGG pathway analysis of gene affected by SB743921 treatment (10 nM, 8 h) in CCLP1 cells. **(C-D)** Flow cytometry analysis of cell cycle distribution of HCCC-9810 and SK-CHA-1 cells following treatment with SB743921 (SB), paclitaxel (PTX), docetaxel (DXT), cabazitaxel (CAB), vincristine (VCR), cephalomannine (CEP), homoharringtonine (HHT) or gemcitabine (GEM) at a dose of 0.5 μM for 24 hours. The histograms showed the fraction of cells in cell cycle phases.

**Fig. S5. Anti-CCA effects of KSP inhibitor SB743921**.

**(A)** Representative images and quantification of c-Caspase3 (c-Cas3) and TUNEL staining in TFK1 CDX models. **(B)** Representative images and quantification of TUNEL staining in HuCCT1 CDX models. **(C)** Representative images and quantification of TUNEL staining in SK-CHA-1 CDX models. Scale bars, 100 μm. Error bars represent mean ± SD, and statistical analysis was performed using the two-sided unpaired *t* test. **p < 0.01, ***p < 0.001, ns, not significant. **(D)** Intratumoral uptake of SB743921 and PTX in CCLP1 CDX models. Intratumoral drug content of CCLP1 CDX models was detected after 1 h with SB743921 (5 mg/kg) or PTX (10 mg/kg) treatment, by Liquid Chromatography Mass Spectrometry analysis. **(E)** Weight of TFK1, HuCCT1, and CC41 xenograft-bearing mice in the drug treatment study.
