# Supplementary material for: The therapeutic effect of KSP inhibitors in preclinical models of cholangiocarcinoma
Source: Cell Death Dis. 2022 Sep 19;13(9):799. doi: 10.1038/s41419-022-05247-0 (PMC9485230; doi:10.1038/s41419-022-05247-0)
Supplement: Supplementary file 9 — Original Data File [file 41419_2022_5247_MOESM9_ESM.pptx]

## Slide 1
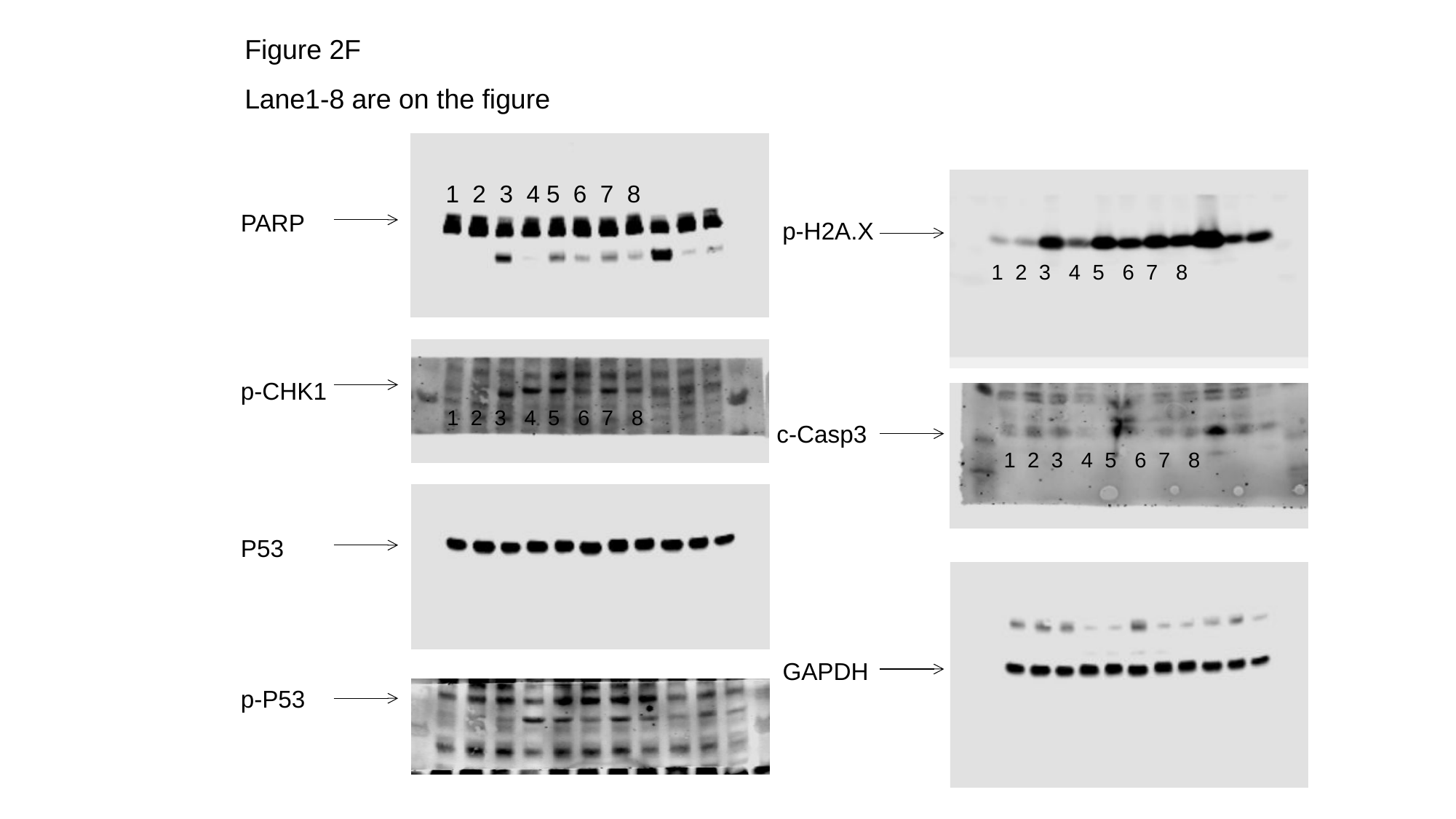

Figure 2F
Lane1-8 are on the figure
1 2 3 4 5 6 7 8
PARP
p-CHK1
1 2 3 4 5 6 7 8
P53
p-P53
p-H2A.X
1 2 3 4 5 6 7 8
c-Casp3
1 2 3 4 5 6 7 8
GAPDH

## Slide 2
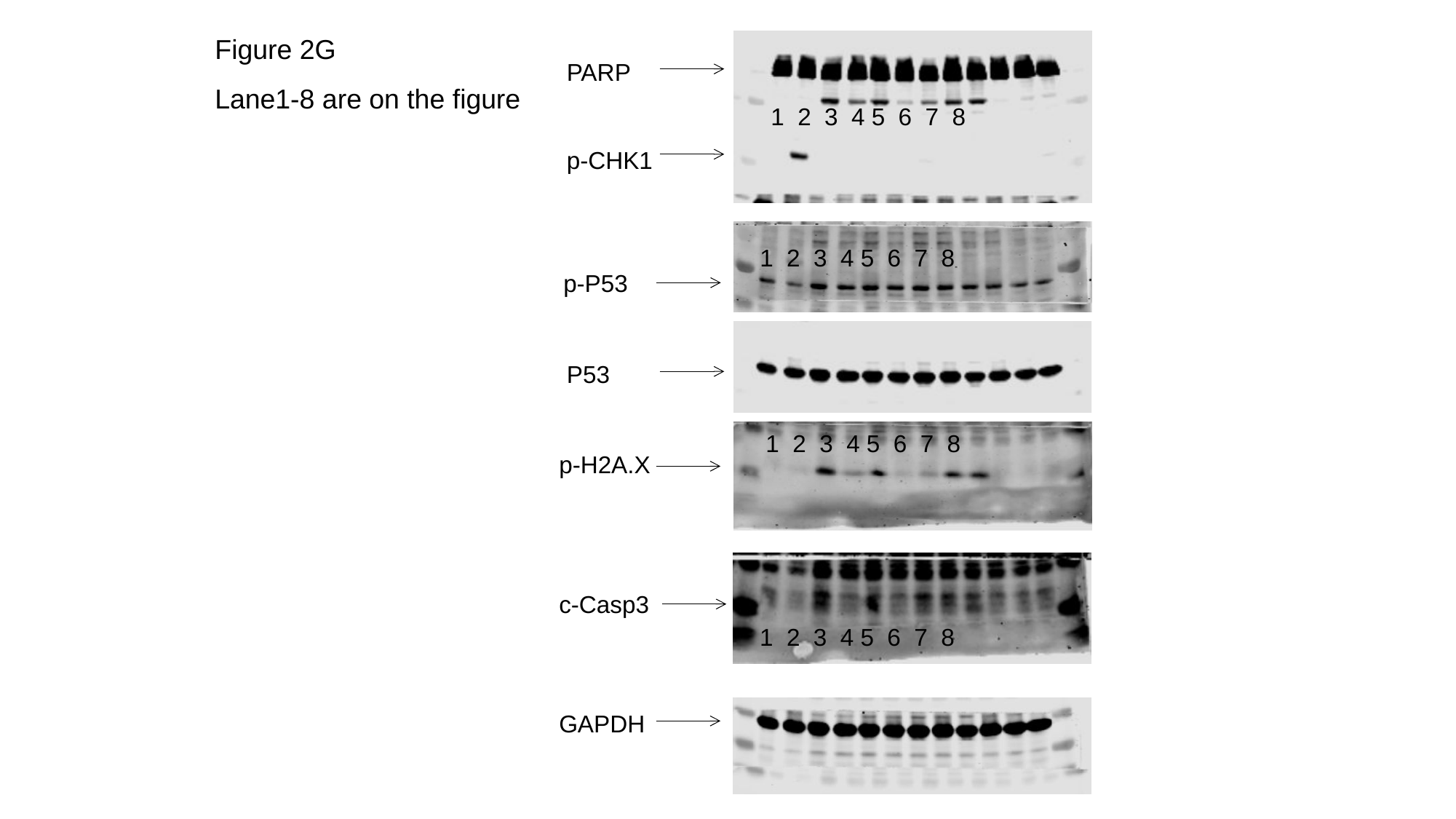

Figure 2G
Lane1-8 are on the figure
PARP
1 2 3 4 5 6 7 8
p-CHK1
1 2 3 4 5 6 7 8
p-P53
P53
1 2 3 4 5 6 7 8
p-H2A.X
c-Casp3
1 2 3 4 5 6 7 8
GAPDH

## Slide 3
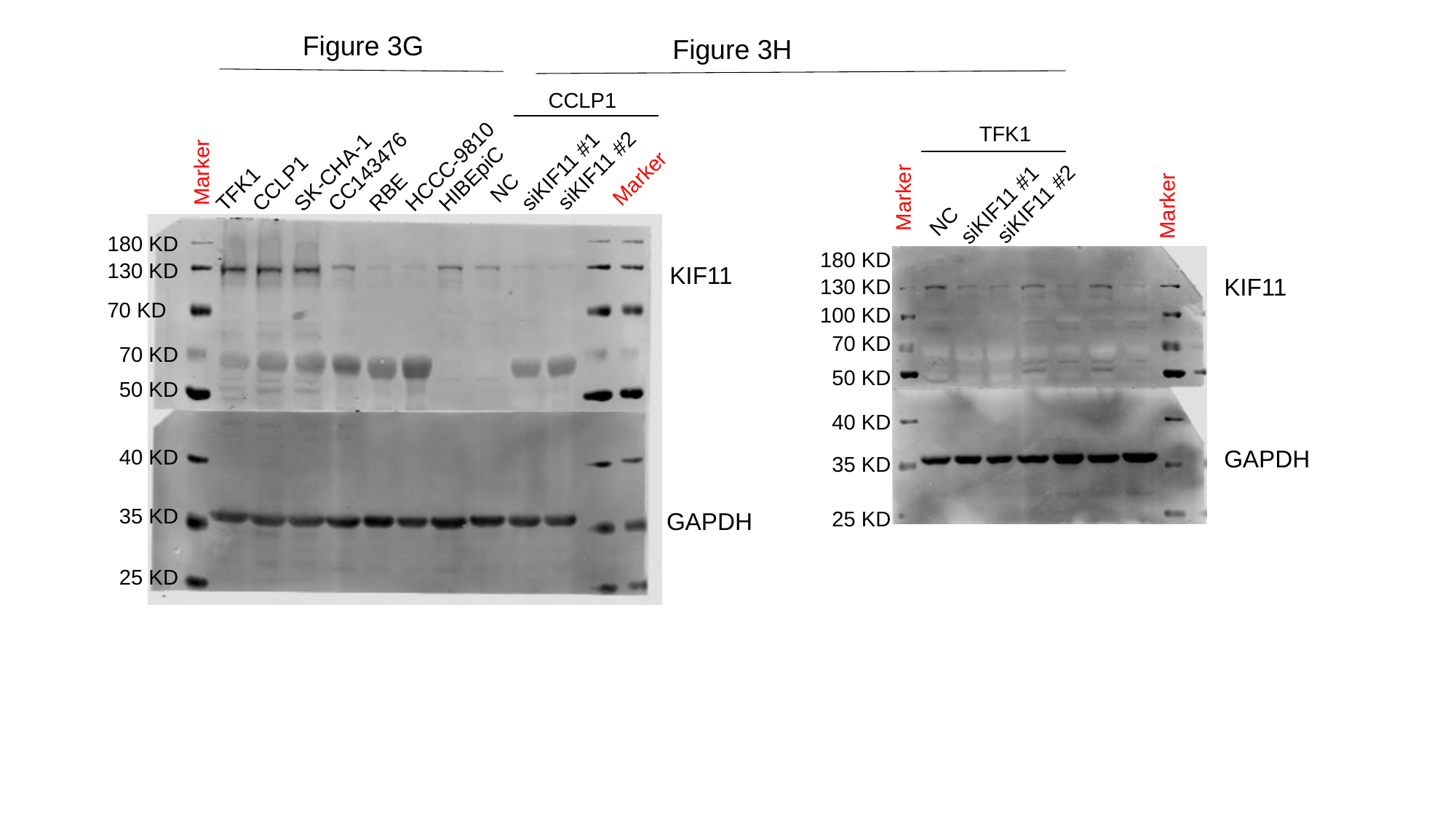

Figure 3G
Figure 3H
CCLP1
TFK1
HCCC-9810
siKIF11 #2
siKIF11 #1
CC143476
SK-CHA-1
Marker
Marker
HIBEpiC
CCLP1
NC
TFK1
RBE
Marker
siKIF11 #2
siKIF11 #1
Marker
NC
180 KD
180 KD
130 KD
KIF11
KIF11
130 KD
70 KD
100 KD
70 KD
70 KD
50 KD
50 KD
40 KD
40 KD
GAPDH
35 KD
35 KD
25 KD
GAPDH
25 KD
